# In-depth patient-specific analysis of tumor heterogeneity in melanoma brain metastasis: Insights from spatial transcriptomics and multi-region bulk sequencing

**DOI:** 10.1016/j.tranon.2025.102468

**Published:** 2025-07-15

**Authors:** Nidhi Sharma, Jana Rájová, Georgios Mermelekas, Kim Thrane, Joakim Lundeberg, Alia Shamikh, Sofi Vikström, Haris Babačić, Margret Jensdottir, Janne Lehtiö, Maria Pernemalm, Hanna Eriksson

**Affiliations:** a Department of Oncology-Pathology, Karolinska Institute, 171 77 Stockholm, Sweden; b Molecular Neuromodulation, Department of Experimental Medical Science, Lund University, 221 00 Lund, Sweden; c Department of Gene Technology, KTH Royal Institute of Technology, 114 28 Stockholm, Sweden; d Science for Life Laboratory, Tomtebodavägen 23, 171 65 Solna, Sweden; e Clinical pathology and Cancer diagnostic Center, 171 76 Solna, Sweden; f Theme Cancer, Skin Cancer Center, Karolinska University Hospital, 171 77 Stockholm, Sweden; g Department of Clinical Neuroscience, Karolinska Institutet, 171 77 Stockholm, Sweden; h Department of Neurosurgery, Karolinska University Hospital, 171 76 Solna, Sweden

**Keywords:** Melanoma brain metastases, Immunotherapy, PD-1, Targeted therapy, Immune signaling, Proteomics, Transcriptomics, Multiomics

## Abstract

Melanoma brain metastases (MBM) exhibit extensive intertumor and intratumor heterogeneity (ITH), driven by a complex tumor microenvironment. The aim of this study was to perform a detailed analysis of individual MBM patient tumors using a multiomics approach, integrating spatial transcriptomics with multi-region bulk exome, proteome, and transcriptome profiling for a small group of four patient samples. We identified significant patient-specific variations in immune cell infiltration, particularly in B/plasma cells, myeloid cells, and cancer-associated fibroblasts (CAFs). Notably, immunotherapy-treated patients showed enriched pathways related to epithelial-mesenchymal transition (EMT), interferon-gamma (IFN-γ) signaling, oxidative phosphorylation, T-cell signaling, inflammation and DNA damage, which aligned with distinct cellular compositions observed in the spatial analysis. We also uncovered considerable ITH, especially at the protein level, revealing differential expression patterns of key tumor and immune-related markers. The correlation between mRNA and protein data highlighted consistent enrichment of critical pathways across multiomics layers. These findings highlight the molecular and cellular landscape of individual patient MBM, underscoring the importance of addressing tumor heterogeneity in the development of effective therapeutic strategies.

## Highlights

• MBM tumors show significant intertumor and intratumor heterogeneity in cellular composition, gene mutations, and pathway enrichment.
• Therapy-treated tumors (P2, P4) exhibited immune activation, while untreated tumors (P1, P3) showed cold tumor signatures.
• P1 and P4 tumors were enriched in CAFs, correlating with epithelial-mesenchymal transition and angiogenesis pathways.
• Proteomic analysis revealed activation of oncogenic pathways like JAK-STAT, NF-κB, MAPK, and EMT, driving tumor progression.

## Graphical abstract

**Figure:**
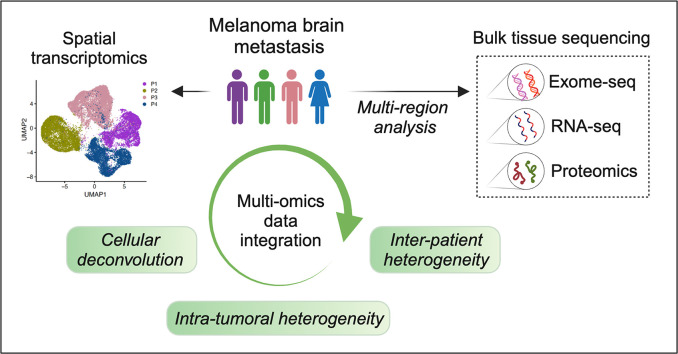

## Introduction

Brain metastases are common in advanced melanoma [1,2], affecting up to 60 % of patients during the course of their disease and accounting for nearly two-thirds of melanoma-related deaths [3]. Prior to the introduction of immune checkpoint inhibitors (ICIs) and targeted therapies, melanoma brain metastases (MBM) were associated with poor outcomes, with a median overall survival of 4 to 6 months [4]. The treatment options were limited to chemotherapy, whole-brain radiation therapy, stereotactic radiosurgery (SRS), and surgical resection [5]. While targeted therapies (such as BRAF, MEK inhibitors) often show rapid initial responses [2], resistance typically develops within 6–12 months [6]. Using ICIs as a first-line treatment has been shown to improve efficacy and prolong survival [7]. In asymptomatic MBM, ICIs have achieved more than 50 % intracranial and extracranial response rates, significantly improving overall survival (OS), though symptomatic MBM continues to exhibit lower response rates and shorter survival [7]. Phase II and III trials of treatment-naive MBM patients have demonstrated significant intracranial efficacy (57–63 % response rates) using combination therapy of nivolumab and ipilimumab, compared to nivolumab alone [8,9]. For BRAF-mutated MBM patients, combining ICIs as first-line therapy with BRAF+MEK inhibitors as second-line therapy has been associated with significantly prolonged OS [2,10]. Systemic therapies, including ICIs (such as anti-cytotoxic T-lymphocyte-associated protein 4 [anti-CTLA-4]—ipilimumab, and anti-programmed cell death protein 1 [anti-PD1]—nivolumab, pembrolizumab) and targeted therapies, sometimes combined with radiotherapy, have improved treatment outcomes in MBM. A multidisciplinary approach, combining surgery, ICIs, and targeted therapies, remains critical for optimizing treatment outcomes [10]. MBM tumors undergo extensive proteomic, transcriptomic, and genomic changes, leading to the emergence of distinct cell populations with diverse phenotypes and functions within the tumor microenvironment [2,11,12]. This complexity, along with both intertumor heterogeneity and ITH, poses significant challenges to the efficacy of current treatment strategies [7]. Malignant melanoma arises from melanocytes under the influence of diverse etiological factors (e.g. ultraviolet radiation and inherited susceptibility) and is characterized by substantial genetic diversity and intratumoral heterogeneity. Emerging evidence also highlights a modulatory role for vitamin D signaling in melanoma progression and treatment response [13].

Over the past few decades, research has primarily focused on defining the genomic landscape of primary melanoma and metastatic disease, uncovering key genetic alterations, such as BRAF, NRAS, AKT1, and PTEN mutations, that shed light on melanoma progression and treatment resistance [3,4,[7], [8], [9]]. Genomic and transcriptomic studies have revealed substantial heterogeneity in malignant melanoma, which complicates the prediction of both prognostic and therapeutic outcomes [4,8,12]. Recent advances in multiomics technologies, including single-cell transcriptomics and proteomics, have provided deeper insights into MBM tumor phenotypes that extend beyond genomic analysis [10,[14], [15], [16], [17]]. Studies utilizing spatial technologies have unveiled the complex multicellular ecosystems of MBM, characterized by spatiotemporal interactions between malignant, immune, and stromal cells [7,14]. Likewise, comprehensive proteomic analyses have been instrumental in profiling the melanoma proteome, identifying diagnostic biomarkers, elucidating molecular pathways of pathogenesis, and understanding therapeutic responses [18]. In-depth proteomic analyses also uncovers alterations that may be overlooked at the genomic level. Exploring the dynamics of tumor tissue and cellular heterogeneity within the MBM microenvironment is crucial for understanding tumor behavior, metastatic potential, and therapeutic responses, ultimately aiding in the identification of effective, personalized treatment strategies. However, to date, there have been limited efforts to fully characterize the molecular and phenotypic landscape of MBM in the individual patients. This lack of integrated multiomics data has hindered a comprehensive understanding of how the spatial organization of the tumor, along with its transcriptome, proteome, and cellular microenvironment, collectively drive tumor behavior and contribute to tissue heterogeneity. Our study aims to address this gap by performing an in-depth analysis of each patient's tumor, integrating spatial transcriptomic analyses with multi-region bulk proteomic and genomic data to provide a more holistic understanding of MBM tumor behavior and its clinical implications.

**Fig. 1:**
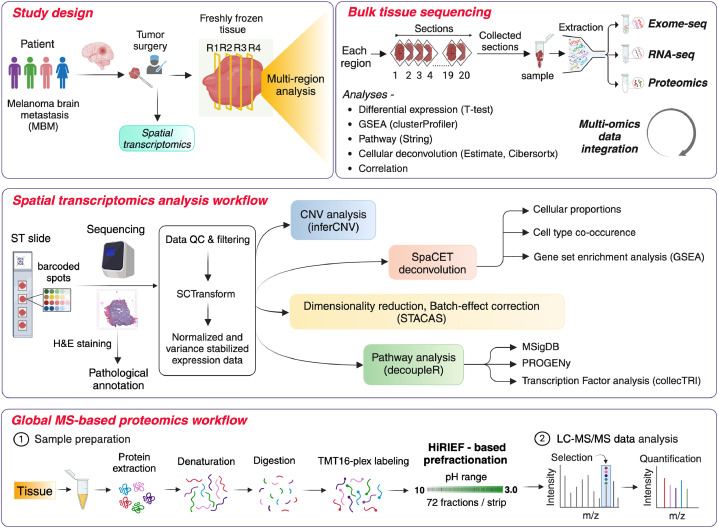
Workflow and multiomics analysis of the melanoma brain metastases. The MBM patient tumor samples were subjected to spatial transcriptomics (ST) and multi-region bulk tissue sequencing. The figure provides a brief overview of the experiment and analysis workflow employed for bulk tissue sequencing, ST and bulk tissue proteomics.

In this study, we address the existing knowledge gap by employing an integrative multiomics approach that combines genomic, transcriptomic, and proteomic data with spatial transcriptomics (ST) to characterize the heterogeneous behavior of MBM in a cohort of four patients (Fig. 1). The small cohort size limits the broader generalizability, instead our study focuses on examining interpatient heterogeneity and delivering a detailed, patient-specific characterization of MBM (Table 1). By integrating multi-layered data, we explore the interactions between genetic mutations, gene expression, and protein dynamics, offering insights into the functional consequences of these molecular alterations. Unlike traditional genomic profiling alone, our approach emphasizes the importance of spatial and cellular heterogeneity both within and between tumors, which is crucial for understanding the complexity of MBM [4,19]. Analyzing multiple tumor regions from each patient allows us to capture both intertumor heterogeneity and ITH, uncovering tumor-associated signaling pathways that may be overlooked by other methods. While these findings are specific to this small group of patients (Table 1), they provide a foundation for future studies with larger, more diverse patient groups to validate these molecular signatures and assess their clinical relevance. Overall, our study highlights the potential of integrating spatial tumor information with multiomics to enhance our understanding of individual MBM tumors and to guide more personalized therapeutic strategies.

**Table 1:** Clinical characteristics of MBM patients.

**Patient (P)**	**Sex**	**Age (years)**	***BRAF V600* mutation**	**Treatment**	**Pathological-anatomical diagnosis (PAD)**	**Survival (post surgery, days)**
P1	male	69	not analysed	stereotactic radiation	necrotic areas present	14
P2	male	76	*BRAF^V600E^*	Nivolumab (anti-PD1) and kinase inhibitors	no necrosis	111
P3	male	64	*BRAF^wt^*	treatment-naive	necrotic areas present	64
P4	female	79	*BRAF^wt^*	Pembrolizumab (anti-PD1)	pigmentation, perivascular tumor growth	223

## Materials and methods

### Ethical approval

The study was approved by the Regional Ethical Review Board in Stockholm, Sweden and by the Swedish Ethical Review Authority and is in accordance with the Declaration of Helsinki.

### Clinical sample collection and processing

We obtained fresh frozen tumor tissues from four patients with MBM. Hematoxylin and eosin (H&E-stained) staining of the tumor tissue was performed by a trained pathologist. The freshly frozen (FF) tissue blocks were OCT-fixed and sectioned using a cryotome in a sequential manner starting with immunohistochemistry (IHC) (7 µm), ST (10 µm), IHC (7 µm), bulk-tumor sequencing (20 µm) and IHC (7 µm). H&E-stained images of each of the four patient tumor samples were manually annotated by a trained pathologist to identify melanoma, stromal and lymphoid tissue. The H&E images and histological description of the tumor are discussed in the supplementary material (Figure S1).

### Patient samples and clinical data

We performed multiregion bulk whole-exome sequencing (WES), RNA sequencing (RNA-seq) and in-depth proteome profiling of four MBM tumor tissues of patients with varying clinical characteristics and treatment regimens (Table 1). MBM patient P1, P2 and P4 tumors had been exposed to treatment prior to MBM surgery. MBM patient 1 (P1) underwent radiation therapy. MBM patient 2 (P2) received both anti-PD-1 and kinase inhibitors, while Patient 4 (P4) had received anti-PD-1 monotherapy. MBM patient 3 (P3) was treatment-naive with *BRAF wild type (wt)* status. The available synoptic pathology information of MBM tumor samples is included in the supplementary material (Table S1). A summary of OS and Progression-Free Survival (PFS) for each patient is provided in the supplementary material (Table S2).

### Visium spatial transcriptomics

#### Preparation of visium spatial gene expression libraries

We performed ST on multiple tumor sections (P1 - 2, P2 - 3, P3 - 3, P4 - 4) using the 10x Genomics Visium platform. Fresh-frozen 10 μm sections were mounted on spatial gene expression slides. Tissue optimization (10x Genomics) was used to adjust permeabilization (staining - 4 min, tissue permeabilization - 18 min). Sections were imaged at × 20 magnification (Metafer Slide Scanning) and stitched with VSlide software. After methanol fixation, H&E staining, and permeabilization, cDNA was generated from mRNA bound to slide capture oligos. Slides were processed, and libraries were prepared per manufacturer’s instructions (CG000239 Rev A, 10X Genomics).

### Bulk-tissue sequencing

#### Clinical sample preparation

Briefly for multi-region bulk sequencing, 20 sequential sections of 20 µm were collected for four regions, starting with regions (R) 1- 4. Each of these regions were separated by an interval of 10 adjacent sections for IHC (7 µm, slide-fixed). The sections collected in tubes were first washed in PBS to remove the blood, centrifuged, and the tissue pellets were used for subsequent DNA, RNA, and protein extraction with the AllPrep DNA/RNA/Protein Mini Kit (Qiagen, Hilden, Germany) as per the manufacturer’s protocol.

#### Whole-exome sequencing (WES): library construction and sequencing

For each DNA sample, concentration and quality were measured using Qubit and Nanodrop. Whole exome sequencing libraries (16 samples) were prepared from 50 ng DNA using the Twist Comprehensive Exome kit (Twist Bioscience) with unique dual indexes, following the manufacturers’ instructions (DOC-001085). Library concentration was evaluated by qPCR, and fragment size distribution was assessed with Tape Station or Fragment Analyzer. After passing QC, libraries were normalized, pooled, and sequenced on an SP flow cell with 100-cycle paired-end 100 bp read length using the NovaSeq 6000 system with v1.5 chemistry.

#### RNA-sequencing (RNA-seq): library construction and sequencing

RNA sample quality and concentration were measured using the Agilent 2100 Bioanalyzer. Ribosomal depletion was performed with Illumina RiboZero GOLD, and RNA libraries were constructed using Illumina TruSeq Stranded Total RNA. Multiplex sequencing was conducted on a NovaSeq6000 (Control Software 1.7.5/RTA v3.4.4) with >50 M reads/sample and 2 × 150 bp pairs, using the 'NovaSeqXp' workflow in 'S4′ mode. Of 16 samples, 4 (P1-R2, P4-R2, P2-R3, P2-R4) failed quality control and were not sequenced.

#### Global proteomics experiment workflow

##### Protein reduction, alkylation, and digestion

Protein samples (eluted in RLT buffer using AllPrep DNA/RNA/Protein Mini Kit) were brought at RT on a heat block (25 °C for 10 min with mixing). For protein clean-up, 250 μl of each sample was processed with Sera‐Mag SP3 beads as described previously [20]. To each sample, 100 μl of lysis buffer (4 % SDS, 25 mM HEPES, 1 mM DTT) was added. Trypsin (1:50 ratio) was used for digestion of 250 µg protein overnight at RT [20]. After digestion, peptides were collected into fresh tubes, and 50 µg per sample was used for TMT labeling. Protein and peptide were measured using the Bio-Rad DC Assay.

##### Tandem mass tags (TMT)-labeling and HiRIEF-based LC-MS/MS

Proteomics experiments were run in triplicates for each sample. For bulk-tissue proteomics, three TMT16plex sets were used, with each set containing four tumor regions per patient (R1, R2, R3, R4), for a total of 16 samples. TMT16plex labeling (Thermo Scientific) was performed according to manufacturer instructions. Labelling efficiency (>95 %) was confirmed by LC-MS/MS using 30-minute gradients. After label validation, samples were pooled and desalted via strong cation exchange (SCX) before LC-MS/MS analysis. The eluted peptides were dried (SpeedVac) and stored at −20 °C.

For HiRIEF prefractionation, the TMT-labeled peptide pool was loaded onto an IPG gel strip (24 cm; linear gradient, GE) for overnight isoelectric focusing. Peptides were extracted into 72 fractions using an in-house IPG extractor robot (GE Healthcare) and freeze-dried. The LC-MS run was performed on a Thermo Scientific Q Exactive-HF coupled to an online 3000 RSLC nano system. The 72 fractions were concatenated to 40 for analysis using a dynamic gradient scheme (Datasheet S1).

### Bioinformatics

#### ST data generation and analysis

##### Visium sequencing and data processing

cDNA libraries quantification was performed using Agilent High Sensitivity Kit (Catalog # 5067–4626) on an Agilent Bioanalyzer 2100. The cDNA libraries were sequenced on a total of 2 flow cells (4 lanes) on Illumina NovaSeq SP-200 with a 28–10–10–90, custom read setup. Demultiplexed FASTQ files were converted to count matrices using the 10x Genomics Space Ranger 1.3.1 pipeline, where the sequencing reads were aligned to the standard GRCh38 reference genome.

##### Data quality control, filtering and analyses

Following the processing of sequencing data, quality control (QC) and normalization was performed on all gene-feature matrices separately. We excluded features with less than 100 transcript or 50 genes, genes present in less than 5 features. The molecule counts were then transformed with SCTransform (Seurat v5.0.1) using 5000 genes and the transcript count per feature was regressed out. All samples were then integrated through STACAS v2.2.2 package, using 1500 anchor features. PCA was then performed with 50 PCs, and a neighborhood graph was constructed, followed by Leiden clustering with a resolution 1.0. CNA was estimated from the transcriptomic data with inferCNV v1.18.1. Adult cutaneous melanocytes (GEO: GSE151091) and brain cells (SPATA2::cnv_ref) were used as reference. Cell cycle was assessed though Seurat’s CellCycleScoring function.

For comparison of expression values between P1-P4 and reference libraries of normal skin cells (GEO: GSE151091) and naive melanoma brain metastases (GEO: GSE185386), reads from all samples were normalize with DESeq2 and then batch corrected with ComBat (ComBat_Seq) with dataset as the batch dimension. The reads were then log-normalized.

##### Deconvolution of ST data

In order to deconvolve the cellular composition of features, SpaCET package (v1.2.0) was used. Malignant proportions was assigned by the package through SpaCET assignment (cancerType = "SKCM”), non-malignant portion was assigned through the remainder of cells were assigned through an external reference (GEO: GSE185386) with main categories: B/Plasma cells, Astrocytes, CAFs, T cells, Myeloid cells, Endothelial cells, Microglia, NK cells, Neurons, Oligodendrocytes and Pericytes according to the original annotation. SpaCET cell colocalization was used to create colocalization graphs. Tumor, interface and stromal areas delineations were used with default parameters.

##### Enrichment analysis transcription factor

Progeny, MSigDB Hallmark and transcription factor (TF) activity analyses were implemented via decoupleR (v2.9.1) through the univariate linear regression model. PROGENy was implemented using 100 top genes per pathway, and MSigDB Hallmark gene sets were used as provided. TF activity scores were inferred from a CollectTRI collection. We set cut-off criteria for p-value significance as *<0.05, **<0.01, ***<0.001 for enrichment analysis. Same procedure was done for two reference datasets (GEO: GSE151091, GSE185386).

#### Bulk-tissue multiomics data processing and analyses

##### Proteomics data acquisition

Database search utilized MSGF+ and Percolator tools in the Galaxy platform to match MS spectra to the human Ensembl version 103 protein database. MSGF+ settings included a 10 ppm precursor mass tolerance, fully-tryptic peptides, a maximum peptide length of 50 amino acids and a maximum charge of 6. Fixed modifications were applied to TMT16plex on lysines and peptide N-termini, along with carbamidomethylation on cysteine residues. Additionally, a variable modification was used for oxidation on methionine residues.

##### Protein quantification, quality control and filtering

Quantification of TMT16plex reporter ions was performed using OpenMS project's Isobaric Analyzer (v2.0) [21]. Each sample was run in triplicates using 3 TMT16plex datasets; the 3 proteomic sets identified 63,880, 66,127 and 70,884 unique peptides respectively, to a depth of more than 8000 unique proteins across all samples (genecentric, PSMs identified at 1 % FDR were used to infer gene identities).

The bulk-tissue proteomic data showed unimodal distribution for the 9699 identified proteins. Among them, 8244 proteins were quantified in at least two of the three TMT sets. To further refine the analysis, proteins with a peptide-spectrum match (PSM) count greater than 1 in at least one of the TMT sets were selected, resulting in a final set of 7732 proteins for analysis. The average coefficient of variation (CV) values among the triplicates in Set 1, Set 2 and Set 3 proteomes were ∼10 to 15 % for each sample respectively. We calculated the average of log2TMT-ratios from the 3 TMT sets for each protein. Gene-level log2TMT-ratio (median normalized) proteomics data containing proteins (*N* = 7732) was used for downstream analysis.

##### Differential proteomic analysis between patients

For proteomic comparisons, differential proteomic analysis was performed on 7732 common proteins that are quantified in all the four patients. Two-tailed paired Student’s T-test was performed to assess the statistical significance, P value of protein expression difference between the patients. We performed differential proteomic analysis between patients - P1 vs. Others, P2 vs. Others, P3 vs. Others and P4 vs. Others, where proteins with BH adjusted P value, FDR < 0.01 and fold change (FC, expressed as ratio of average protein expression ratio between the two groups) > ± 1 were considered to be significantly upregulated or downregulated. For ITH analysis, we performed differential proteomic analysis for multiple neighboring regions within each tumor, where proteins with BH adjusted P value, FDR < 0.05 and log2 fold change (FC, expressed as ratio of average protein expression ratio between the two groups) > ± 0.5 were considered to be significantly upregulated or downregulated.

#### NGS data quality control and analysis

Quality control of all NGS samples (WES, RNA-seq) was performed using FastQC (v0.11.9), FastQ Screen (v0.14.1) [22], MultiQC v1.8 [23] and QualiMap (v2.2.2-dev).

#### WES analysis: alignment, variant calling, and mutation clonality identification

The WES data was analyzed with the Nextflow (v20.11.0-edge) based nf-core/Sarek (v2.6.1) pipeline. In brief, reads from FastQ-files are mapped to a reference genome using BWA (v0.7.17-r1188). Bam-files are de-duplicated with GATK (v4.1.7.0) MarkDuplicates. Base quality score recalibration tables are created with GATK BaseRecalibrator. The tables are then used in GATK ApplyBQSR to create recalibrated bam-files; tools were taken from GATK (v4.1.7.0).

Somatic single nucleotide variations, small insertions and deletions were all detected using Strelka (v2.9.10). Variant calling was done using GATK tools and Bcftools (v1.9) and annotated using SnpEff (v4.3t). After running the pipeline, Picard CollectHsMetrics [24] is used to evaluate the coverage.

#### Exploratory data analysis

We filtered out variants that are too common to be pathogenic using the low max_AF cutoffs of 0.0001 (0.01 %), and remaining variant data was used for further quantitative analysis. Out of the total novel variations identified, the novel variants with Impact status either ´moderate´ or ´high´, and variant frequency of > 5 were analyzed and plotted to extract significant novel gene variants in each tumor region.

#### RNA-seq analysis: alignment and processing

The publicly available nf-core/rnaseq bioinformatics pipeline was used for processing RNA data. The pipeline takes FASTQ files as input, performs quality control (QC), trimming and (pseudo-)alignment, and produces a gene expression matrix and extensive QC report. The nf-core/rnaseq pipeline was run using the following software versions – bedtools (2.29.2), bioconductor-summary (1.20.0), bioconductor-tximeta (1.8.0), deseq2 (1.28.0), dupradar (1.18.0), fastqc (0.11.9), nextflow (20.11.0-edge), nfcore/rnaseq (3.0), picard (2.23.9), preseq (2.0.3), qualimap (2.2.2-dev), rseqc (3.0.1), salmon (1.4.0), samtools (1.10), star (2.6.1d), stringtie (2.1.4), subread (2.0.1), trimgalore (0.6.6) and ucsc (377).

#### Data filtering and quantitative analyses

The RNA data included 37,210 rows after removing rows with zeros across all columns. To further reduce noise, we retained rows with gene counts ≥ 10, in at least half of the samples, resulting in 18,784 gene rows for downstream analysis. For QC, normalization, and quantitative analyses, the data was processed using the DESeq2 pipeline. DESeq2 fits data using the parametric Wald test and normalizes for sequencing depth and RNA composition by generating median ratios (counts divided by sample-specific size factors). Variance-stabilized data was used for PCA and sample-to-sample distance calculations. For untreated vs. treated comparisons, 2037 DEGs were identified, where proteins with baseMean > 50, adjusted P value (FDR) < 0.001 and fold change (FC) > ± 2 were considered significantly up- or downregulated.

#### Deconvoluting cell populations

ESTIMATE was utilized to infer immune and stromal scores [25] and CIBERSORTx was used to estimate the abundance of MBM cell-type specific proteins in the normalized RNA-seq data. Here, the MBM cell-type marker gene profiles generated from the recently published research work by Biermann et al. [26], was used as a reference “signature matrix” to enumerate cell subsets in bulk tumor tissue samples.

#### Differential transcriptomic analysis

For transcriptomic comparisons, the analysis was performed on 7732 common proteins that were quantified in all the patient samples. Two-tailed paired Student’s T-test was performed to assess the statistical significance, P value of protein expression difference between the patients. We performed differential proteomic analysis between patients - P1 vs. Others, P2 vs. Others, P3 vs. Others and P4 vs. Others, where proteins with BH adjusted P value, FDR < 0.01 and fold change (FC, expressed as ratio of average protein expression ratio between the two groups) > ± 1 were considered to be significantly upregulated or downregulated.

#### PPI network analysis

We submitted the list of DEPs to the Search Tool for the Retrieval of Interacting Genes (STRING) database, selecting the highest confidence score (≥0.7, ≥0.9) to create the interaction network. The disconnected nodes with no protein-protein interactions were hidden and excluded from the network analysis. The network included both known and predicted interactions between the input proteins and other proteins in the STRING database. We employed k-means network clustering algorithms to identify the significantly enriched clusters or modules. Statistical significance was assessed using multiple testing corrections, FDR < 0.05. The nodes with >6 connections were considered as the key hub proteins.

#### Functional annotation

We used MBM cell-specific markers and Molecular Signatures Database (MsigDB) gene sets for analysis [26,27]. Gene set enrichment analyses (GSEA) was performed to calculate enrichment scores for gene profiles based on ontology gene sets from the MsigDB C5 collection. The normalized enrichment score (NES) quantified gene enrichment, with positive NES indicating genes enriched at the top and negative NES indicating those at the bottom of the ranked list. R packages, including clusterProfiler and GSEA, were used to infer dysregulated KEGG pathways and Hallmark gene sets enriched in differential protein sets, such as (1) up-/downregulated proteins in untreated vs. treated samples, (2) region-based comparisons within tumors. Statistical significance (P value) was evaluated using permuted feature test statistics and adjusted with BH correction, with significant gene sets identified at a q value < 0.05.

#### Statistical analysis

All data visualizations were produced with R version 4.3.3 in RStudio. The r packages used for data analysis and plots were made using the r packages, including ggplots, dplyr, tidyverse, msigdbr, org.Hs.eg.db, clusterProfiler, pheatmap, GSEABase, biomaRt, easypackages, WGCNA and cowplot. Spearman's correlation was used to compute the correlation coefficient. Two-tailed paired Student's *t*-test was used to compute significant differences in expression to identify up or down-regulation in proteomics and transcriptomics.

## Results

### Spatial transcriptome mapping reveals the MBM tumor tissue organization and cell-type composition inference

**Fig. 2:**
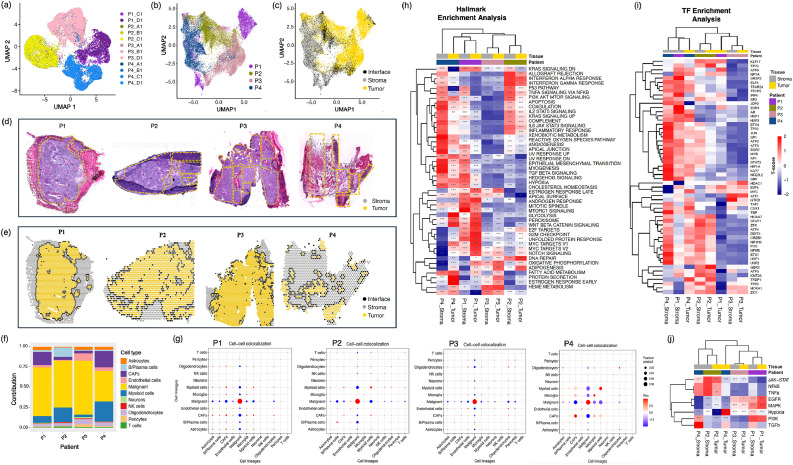
Spatially-resolved transcriptomics reveal tumor heterogeneity and cellular diversity in MBM patients. (a) UMAP projection of non-batch-corrected, regressed ST data spots aggregated from 12 spatially-profiled samples, spots colored based on patient samples (≥ 2 replicates for each patient). UMAP embedding of (b) normalized and batch-corrected spots, and (c) deconvoluted spots based on the tumor, stroma and interface gene expression profiles. H&E staining of cryosections used for ST. (d) Pathological annotations, and (e) spatial distribution of tumor, stroma and interface annotated spots. (f) Stacked bar plot showing the fractional composition of cell number within the tissue section of all patient samples. (g) Correlation plot of all cell types within the major tier for each patient tumor. Here, cell-cell colocalization scores are indicated by Rho. (h-j) Heat map displaying cancer hallmark, averaged transcription factor (TF) activity and averaged PROGENy results for all the patient samples. Here, the significance levels are as mentioned: *<0.05, **<0.01, ***<0.001. results for all the patient samples. For TF heatmap representation, significance cut-off of < 0.001 was used to visualize TFs with high statistical significance in the heatmap. Scale numbers in legend represent the mean t-score in that sample type, with positive scores (red) indicating increased activity and negative scores (blue) indicating decreased activity.

We conducted spatial tumor profiling to infer the cellular organization and molecular landscape of each MBM tumor tissue. The spatial gene expression data underwent quality control, normalization, batch effect correction, and dimensionality reduction for subsequent analysis (Fig. 2a-c; Figure S2–3). The malignant tumor, stroma and interface spots/features identified within tissues using ST, correlate well with H&E-staining and morphological regions annotated by the pathologist (Fig. 2d,e). Next, we deconvoluted non-malignant cell types present in the tumor microenvironment [28], confirming the presence of malignant, B/Plasma, myeloid, stromal, endothelial, T/NK and CNS cell types (Figure S4). Significant intertumor differences were observed across all the four tumors, with P2 and P4 specifically enriched in myeloid, B/Plasma, and T cell types. Notably, patients P1 and P4 showed enrichment in the abundance of CAFs (Fig. 2f). Cell-cell correlation analysis revealed metastasis-specific cellular neighborhoods within each MBM tumor, such as a distinct correlation observed between malignant cells, B/Plasma, CAFs and myeloid cells (Fig. 2 g). The ST data findings are in line with recently published research studies that highlight intricate spatiotemporal interactions between malignant, immune, and stromal cells, further emphasizing the dynamic nature of the tumor microenvironment and complex multicellular ecosystems of MBM [29,30].

To further assess intertumoral heterogeneity, we analyzed the activations of key oncogenic pathways, and transcriptional activities related to tumor progression across all patients. While systemic therapy-treated tumors, P2 and P4, were enriched in JAK-STAT, NF-κB and TNF-α pathways, P1 and P3 tumors showed enrichment in EGFR and MAPK signaling (Fig. 2h). Both P1 and P4 tumors exhibited elevated gene expression related to epithelial-mesenchymal transition (EMT) and angiogenesis (Fig. 2i), which aligns with the significant enrichment of CAFs observed in the cellular deconvolution of the spatial transcriptome for these patients. Cellular function hallmarks, such as IFN-α, IFN-γ, IL6-JAK-STAT3, inflammatory, coagulation, and complement signaling, were enriched particularly in systemic therapy-treated P2 and P4 tumors. In comparison to treatment naive patient P3, all treated tumors (P1, P2, P4) displayed significantly higher expression of Il2-STAT5, KRAS and ROS signaling pathways.

With CollectTRI database, we used a univariate linear model to infer TF activity and identified unique TF clusters related to tumor progression in each tumor. For example, the top-enriched TFs in treatment-naive patient P3 included MITF, TBPL1, OTX2 and PLAG1 (Fig. 2j). Differential patterns of cancer hallmark, cellular pathways and TFs highlight intertumoral heterogeneity, treatment-modulated immune responses, and their tumor regulating roles within the MBM tumor microenvironment.

### Validating ST findings through comparison with normal melanocytes and naïve MBM

We compared the molecular profiles of the four MBM samples (P1–P4) with two reference datasets: melanocytes from surgical cuttings and treatment-naive melanoma brain metastases (Figure S5). This revealed distinct patterns, with P2 and P4 (immunotherapy-treated) showing immune activation, while P1 (radiotherapy-treated) and P3 (treatment-naive) were cold tumors, aligning with naïve melanoma cells. Pathway analysis highlighted MAPK and EGFR activation as key differentiators, elevated in P1 and P3 but suppressed in P2 and P4, reflecting therapy-driven changes. Hypoxia scores varied across metastases, indicating microenvironmental heterogeneity (Figure S5). Hallmark gene set analysis identified a proliferation module active in P1 and naïve tumors, while metabolic pathways were broadly activated across all tumor cells. CAFs shared damage responses with melanocytes, indicating convergent stress adaptation. TF activation patterns aligned P1 and P4 with CAFs, and P3 with naïve melanoma cells. MYC was upregulated in all tumor regions, but MITF activity varied—absent in P1 (dedifferentiated) and elevated in P3 and P4 (melanocytic). P2 displayed intermediate MITF levels, suggesting a transitional state (Figure S5). These findings illustrate unique survival strategies across MBM samples, with P2 and P4 showing immune activation, while P1 and P3 lost melanocytic identity. CAFs maintained consistent pathway signatures across metastases, indicating stable tumor-stromal interactions. Further, CNV analysis also identified high tumor cell content in specific regions, highlighting genomic alterations across patients (Figure S6). In summary, ST analysis revealed significant intertumor heterogeneity in cellular composition, pathway enrichment, and TF activity within MBM patient tumors. Spatial arrangement and interaction patterns between cell types reveal patient-specific and treatment-specific tumor features, consistent with tissue morphology and pathologist annotations.

### Characterizing MBM tumor heterogeneity through multi-region bulk sequencing

To understand the underlying molecular mechanisms in MBM, the spatial transcriptomic mapping of the MBM tumor tissue was complemented by multiregion bulk genome, transcriptome, and proteome tissue sequencing. Our aims were to examine tumor tissue organization, intertumor heterogeneity and intratumor heterogeneity (ITH) revealed by the spatial transcriptomic analysis, within the tumor tissue of all four patients (Fig. 1). Four distinct tumor regions were analyzed for each MBM tumor, enabling analysis of geographically distinct regions and facilitating ITH assessment. We further integrated the multiomics data to get an in-depth overview of the cellular and molecular composition of MBM tissue, revealing patterns of brain metastasis in advanced metastatic melanoma.

### Multi-region exome profiling highlights genomic heterogeneity in MBM

**Fig. 3:**
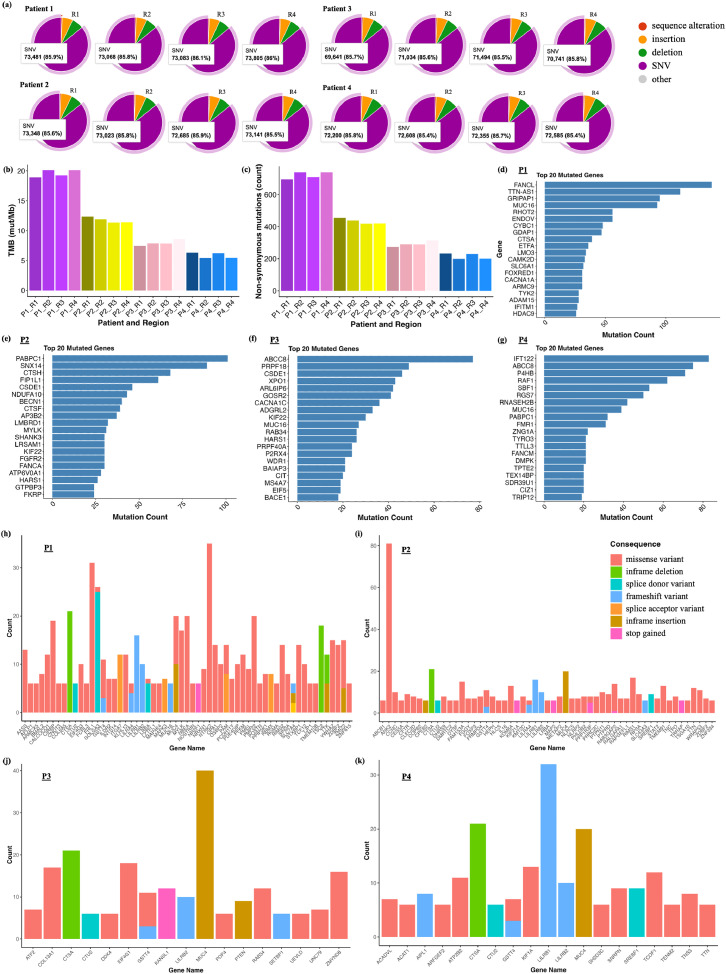
Multi-region whole exome sequencing (WES) of MBM. (a) Summary and proportion of overall consequences ( %) identified in each tumor sample. (b) The comparison of tumor mutation burden, and (c) total non-synonymous mutations identified in each sample. Bar plots displaying the number and types of variants identified for multiple regions within each tumor are provided in the supplementary Figure S7. (d-g) Shows top 20 mutated genes found in each sample. (h-k) Potential novel gene variants identified in each tumor. Here, we have shown representative plots for each patient tumor region in mutation and novel variants plots (d-k). Individual plots for mutation and novel variants identified in multi-region tumor analyses are provided in the supplementary Figures S7–8.

We generated WES data from multi-region bulk-seq of each patient tumor as previously described in Fig. 1. WES data summary shows the number of somatic gene mutations and other variants found in each tumor (Fig. 3a). For clinical significance, we filtered variants with MAX_AF ≤ 0.0001 (0.01 %) to focus on rare mutations, which are more likely to be somatic and relevant to cancer. The proportion of different non-synonymous mutations (e.g., missense, nonsense, frameshift, splice region mutations) in each tumor, is provided in Figure S7. Tumor mutation burden in MBM patients varied greatly between patients, ranging from 5.4 to 20.1 mut/mb. The highest tumor burden was observed in patient P1, who had received radiation therapy prior to tumor resection, followed by patient P2, who had received combined systemic therapy. The tumor burden was lowest in patients P3 and P4. No significant changes were observed in tumor mutation burden of multiple regions within the tumor (Fig. 3b). Non-synonymous mutations varied significantly in all patients, exhibiting significant intertumor heterogeneity. We found an average of 415 non-synonymous mutations (range 200 – 740) for each patient tumor (Fig. 3c). All mutation types identified in multiple regions of each tumor sample, including missense mutations, in frame deletions/insertions, frameshift variants, splice region variants, synonymous and other mutations, are shown in Figure S7. The mutational signature observed in all the four patients was similar to that observed in the TCGA- pan cancer whole genome analysis of metastatic melanoma (MM) patient cohort [11,31]. The distribution of genetic mutations in common key oncogenic signaling pathways, identified oncogenic, likely pathogenic missense variants in several genes, including MYC (MYCL), TP53 (MDM4, MDM2, ATM), Cell cycle (CDK2NA, EGFR), NOTCH (NOTCH3, NOTCH1, MAML3, CNTN6), HIPPO (FAT4), WNT (LGR5, DVL2) and RTK-RAS (KSR1). Next, we looked at the top mutated genes with high variants frequency across multiple regions in each tumor (Fig. 3d-g; Figure S8). Patient tumors P2, P3 and P4 were particularly enriched in gene variants associated with the RTK-RAS signaling pathway, consistent with findings from large clinical cohort studies on the genomic and transcriptomic landscapes of MM [28,32,33]. While patient P2 had significant gene mutations related to MAPK and inflammasome-related signaling, the patient P4 included significant gene mutations (EGFR, ACE, TOP2A, PTGER4, COL18A1, and MMP12) associated with the EGFR pathway. In addition, we found several novel gene variants in multiple regions of each patient tumor (Fig. 3h-k, Figure S9). Most of these variants are common in MM [31,33], highlighting the need for deeper clinical investigation using multiomics analysis to devise personalized treatment strategy for MBM patients.

### Bulk tissue proteome and transcriptome profiling

**Fig. 4:**
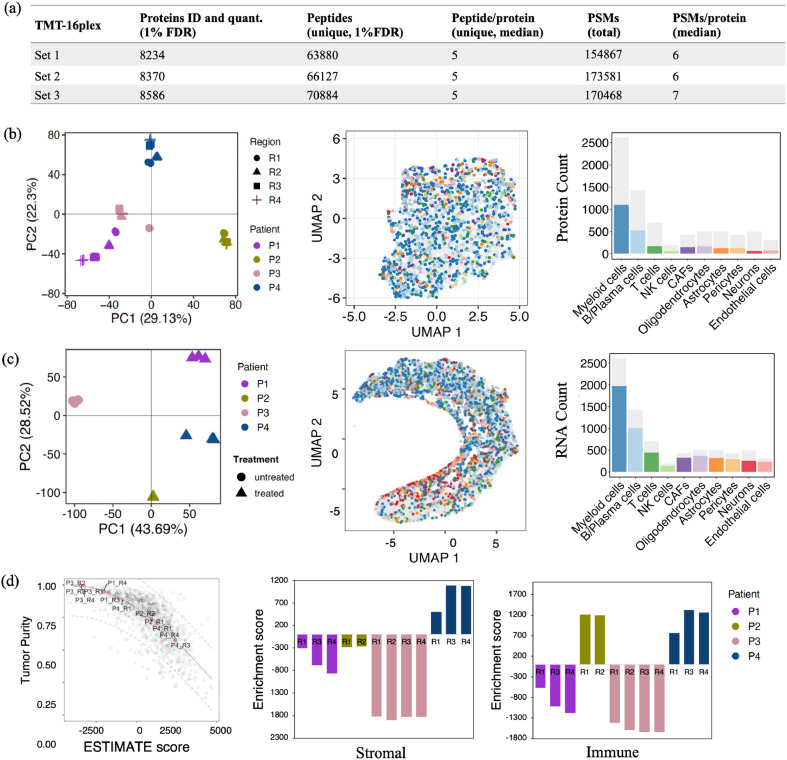
Multiregion bulk proteomic and transcriptomic analysis of human MBM. (a) Summary of MS analysis showing the detected MBM proteome, highlighting the number of proteins identified and their abundance across samples. (b, c) Principal component analysis (PCA) and Uniform Manifold Approximation and Projection (UMAP) plots, were used to visualize the variance and cell-type composition within the samples for the proteome and transcriptome. UMAP projection and histogram showing cell-type distribution based on protein and gene expression, with color-coded annotations in the legend corresponding to the identified cell types. (d) Estimation of tumor purity, stromal content, and immune cell infiltration using the ESTIMATE algorithm applied to gene expression data from the transcriptomic analysis. The plots provide a quantitative assessment of the non-malignant cellular components and immune infiltration in each tumor region and variation across samples.

**Fig. 5:**
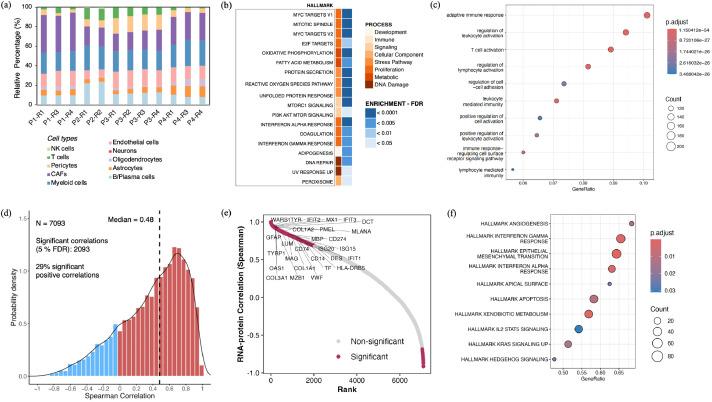
Comprehensive transcriptome analysis of MBM with cell deconvolution, pathway enrichment, and correlation assessment. (a) Deconvolution of tumor cell populations in MBM transcriptome using CIBERSORTx. (b) Enrichment of hallmark pathway-related proteins, and (c) GO functional enrichment analysis of the transcriptome. (d) Venn diagram showing overlap between the MBM proteome and transcriptome. (e) Correlation plot displaying significant correlations and their statistical significance using Spearman correlation. (f) Cancer hallmark pathway enrichment for co-detected genes, with adjusted p-values (FDR) used for significance.

The MBM tissue proteome and transcriptome analysis quantified 7732 proteins and 18,784 protein‐coding genes, respectively (Fig. 4a). Principal component analysis (PCA) revealed a distinct separation between patient tumors based on both protein and gene expression data, with each tumor forming its own distinct cluster. Notably, additional variance was observed within different regions of the same tumor at the protein level (Fig. 4b,c). To determine whether bulk data could capture cellular compositions identified in the ST analysis, we evaluated MBM cell type-specific signatures and enriched molecular pathways across all tumor samples. While UMAP embedding and clustering of protein and gene expression data did not detect major cell-specific clusters (Fig. 4b,c), there was significant global enrichment of myeloid and B/Plasma cells in both the proteome and transcriptome. Consistent with the ST analysis, systemic therapy-treated patients (P2 and P4) exhibited high immune cell infiltration scores (Fig. 4d), with P4 also showing a positive stromal enrichment score. Furthermore, immune-cell deconvolution revealed notable differences in the distribution of myeloid cells, CAFs, B/Plasma cells, and T cells across the patient tumors (Fig. 5a). Importantly, cellular deconvolution findings from the bulk multiomics data aligned with the spatial cell-type distributions observed in the ST analysis (Fig. 2). The MBM also showed significant enrichment in 18 hallmark pathways, including those related to proliferation, metabolic, stress, signaling, immune function, and DNA damage (Fig. 5b). Similarly, the MBM transcriptome showed enrichment for several immune signaling pathways, as seen in the Gene Ontology–Biological Processes (GO-BP) analysis (Fig. 5c).

### Correlation between proteome and transcriptome

To further explore the agreement between transcriptome and proteome analysis, we compared the mRNA and protein expression among the 7093 co-detected genes and calculated gene-wise mRNA:protein correlations across tumors. The median gene-wise correlation was 0.48, with 29 % of genes showing statistically significant positive correlations (5 % FDR) (Fig. 5d). The ranked correlation distribution highlights significantly correlated markers specific to MBM cell types, including interferons (IFN), T-cells, fibroblasts and MHC-II (Fig. 5e). However, a substantial portion of genes did not show strong correlation, which may be attributed to post-transcriptional and post-translational regulatory mechanisms, such as alternative splicing, mRNA stability, or post-translational modifications (PTMs) like phosphorylation or ubiquitination [10]. These mechanisms can alter protein levels, activity, and function independent of mRNA expression, contributing to the observed discordance between transcript and protein levels. Additionally, protein degradation rates, localization, and other factors like microRNA regulation may also play roles in limiting mRNA-protein correlation [15]. Taken together these alterations highlight the importance of multiomics data analysis. Despite these discrepancies, both the proteome and transcriptome analyses consistently revealed enrichment for IFN-γ, EMT, cell proliferation, and key immune cell signaling pathways in each patient tumor, as observed in the ST data (Fig. 5f).

### Determining intratumor heterogeneity using multiregion proteomic data

To determine the levels of ITH within MBM, we characterized protein expression patterns for multiple neighboring regions within the tumor using multi-region bulk proteomic analysis. PCA plot and heatmap representation shows unique variance distribution and protein expression of multiple regions within each tumor (Figure S10–11). To identify ITH present within each tumor, we performed DE analysis (statistical significance criterion: FDR < 0.05, log2FC > ±0.5) to make individual comparisons between the regions on the proteome level. Multi-region proteomics confirmed different levels of heterogeneity observed across multiple regions in each tumor (Figure S10). The DEPs identified for multi-region ITH analysis of each patient tumor varied significantly in terms of proteins linked to MBM cell types, particularly myeloid, B/Plasma, myeloid, stromal, CAFs, endothelial and CNS cells. We further conducted GSEA- Hallmark analyses to elucidate the biological relevance of DEPs and their potential roles in the ITH observed within the patient tumor. The analysis revealed significant protein enrichments in hallmark pathways including MYC targets, IFN-γ, EMT signaling, oxidative signaling, TNF-α, KRAS, IL2-STAT5, MTORC1, and the Complement system (Figure S11). This shows that tumor-driving pathways and cellular composition varies within each MBM tumor, though to a lesser extent compared to between individual patients.

### Proteome-level analysis of intertumor diversity

**Fig. 6:**
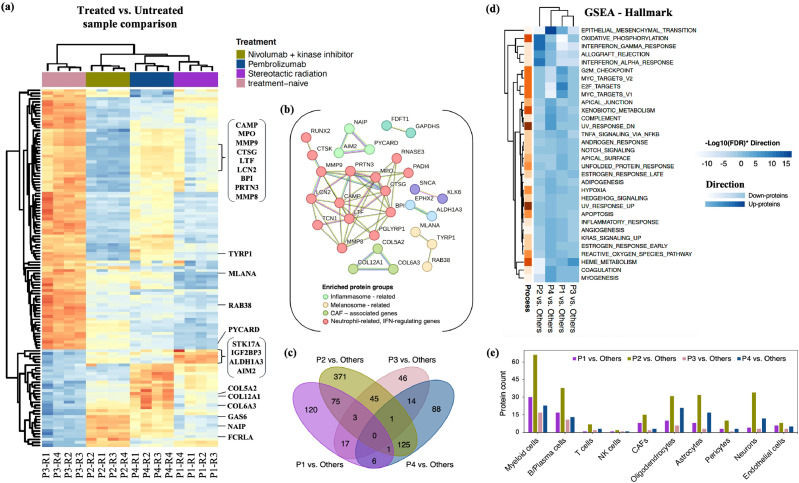
Intertumor proteomic differences in MBM. (a) Hierarchical clustering of differentially expressed proteins (DEPs) from Treated vs. Untreated patient comparisons. (b) String PPI network (confidence level – 0.7) for DEPs, highlighting clusters related to inflammasome, melanosome, CAFs, and IFN-related proteins. These clusters are reflected in the heatmap. Results from DEPs for comparisons of P1 vs. Others, P2 vs. Others, P3 vs. Others and P4 vs. Others. (c) Venn diagram showing the overlap of DEPs with altered abundance in each patient tumor compared to others. (d) GSEA of MsigDB hallmark gene sets, with heatmap displaying enrichment of 50 gene sets (FDR < 0.05) for each tumor, colored by -log10(FDR)*Direction. Here, direction corresponds to negative and positive normalized enrichment score (NES) depicting up-regulated and down-regulated proteins. (e) Enrichment of MBM cell-specific proteins among DEPs identified in interpatient comparisons (DEP cut-off: log2FC > ±1, FDR < 0.01).

To study patient-specific differences, we quantified protein expression patterns across tumors to assess intertumor heterogeneity. Hierarchical clustering demonstrated that each patient tumor region clustered distinctly from the others, indicating greater intertumor variability than intratumor variability at the protein level. Patients P1 (radiation-treated) and P3 (treatment-naive) clustered closely together, while P2 and P4, who received systemic therapy, formed a separate cluster—consistent with the shared cell types and pathways observed in ST analysis (Fig. 6). For treated versus untreated tumor comparisons, proteins with a fold change > ±1 and FDR < 0.01 were considered statistically significant. We identified 123 differently expressed proteins (DEPs) between treated versus untreated tumors that exhibited strong co-expression patterns, as illustrated by protein-protein interaction (PPI) network clusters. These clusters included neutrophil-related genes, IFN-γ regulatory proteins, and proteins associated with melanosomes and inflammasomes (Fig. 6a,b).

Next, we compared the proteome of each patient relative to the others, identifying 222 DEPs in P1, 621 in P2, 126 in P3 and 235 in P4, respectively (Fig. 6c, Figure S12). Gene set enrichment analysis (GSEA) for hallmark gene sets revealed distinct protein profiles between patients. Notably, P2 and P4 tumors showed elevated EMT signaling compared to other patients. Patient-specific DEPs in these patients were also enriched for proteins involved in DNA damage repair, T-cell signaling, IFN-γ response, and drug metabolism, aligning with observations from ST analysis (Fig. 6d). DEPs across all patient comparisons were significantly enriched for proteins associated with myeloid, B/Plasma and CNS cell types —consistent with ST data. These proteins included growth factors, innate immune proteins, melanoma-associated antigens, and TFs involved in B-cell and T-cell development, lymphocyte recruitment, and receptor signaling (Fig. 6e). Additionally, GSEA using KEGG pathways revealed significant intertumor functional enrichments for proteins related to ribosomes, focal adhesion, OXPHOS, ECM-receptor interaction, and cell adhesion molecules (CAMs) across all tumors (Figure S13).

### Network and protein-protein interaction analysis reveals key functional clusters in MBM tumors

**Fig. 7:**
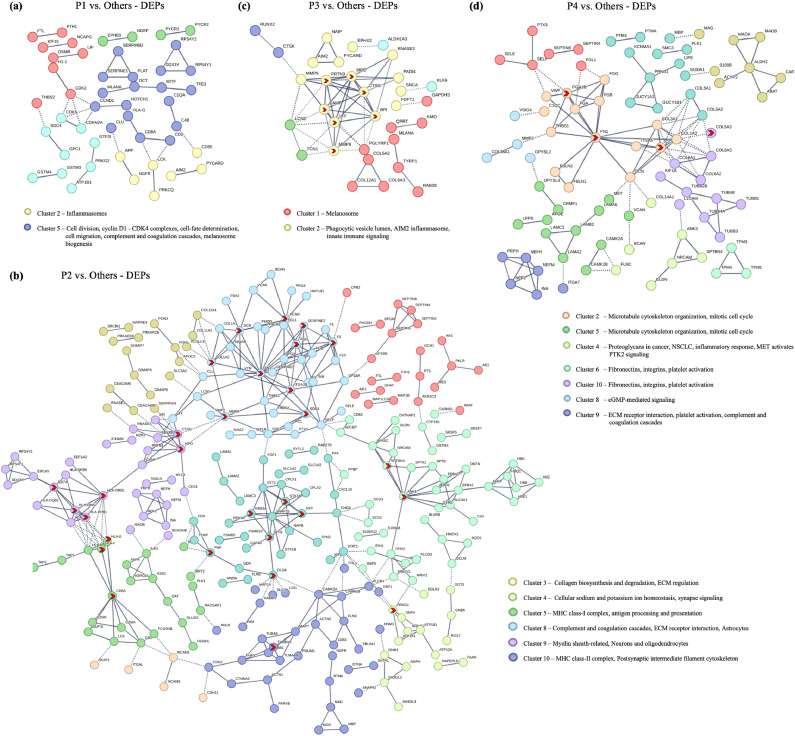
Protein-Protein interactions (PPI) networks for DEPs in MBM. PPI networks for DEPs identified in each patient comparison: (a) P1 vs. Others, (b) P2 vs. Others, (c) P3 vs. Others and (d) P4 vs. Others. The networks display protein interactions with a confidence score > 0.7 or 0.9, with proteins clustered using k-means. Each node represents a protein, and the thickness of the edges indicates the interaction confidence. Disconnected nodes are not shown. Legends highlight key protein clusters within each network.

To investigate the functional associations among DEPs across individual MBM tumors, we conducted a protein-protein interaction (PPI) analysis using the STRING database. We excluded the disconnected nodes to get clearer insights into network organization and functionally enriched clusters that highlight key biological pathways corresponding to tumor development and progression in MBM tumors [34,35] (Fig. 7). In the PPI network analysis, distinct functional clusters were identified across MBM patients, highlighting key proteins and pathways linked to cancer progression. For P1, clusters enriched in ER lumen-associated proteins (CIP2A, OSMR, WWTR1), inflammasome-related proteins, and melanoma-specific proteins (CCND1, MITF, MLANA, NOTCH1) were notable. In P2, a larger network included clusters enriched in enzymatic activity, neuronal tumor suppressors (CDH11, ITGAL), and T-cell signaling (HLA, CD2, LCK, ZAP70). For P3, clusters were enriched in melanosome biosynthesis (TRYP1, MLANA), immune regulators (AIM2, PYCARD), and ECM proteins. In P4, key clusters involved the PI3K-Akt signaling pathway, ECM-receptor interactions, and complement-coagulation cascades, with additional enrichment in metabolism-related proteins and NSCLC markers (MET) (Fig. 7). The network findings emphasize critical biological processes involved in melanoma progression, with particular enrichment in proteins related to inflammation, apoptosis, T-cell signaling, and melanoma-specific pathways. The identification of key hubs and enriched clusters adds valuable insights into the underlying biology of MBM and motivates further investigation into their roles in MBM progression.

### Transcriptome-level analysis of intertumor diversity

**Fig. 8:**
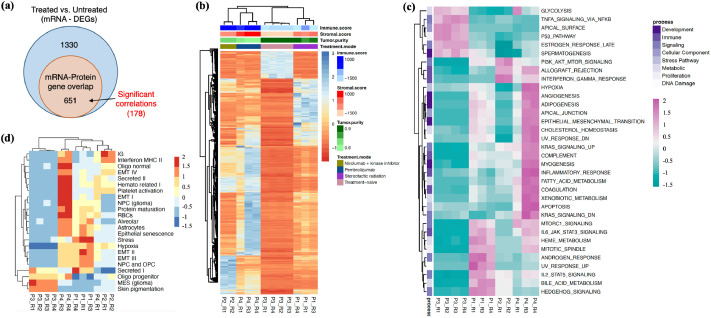
Intertumor transcriptomic differences in MBM. (a) Total number of DEGs identified in treated vs. untreated tumor comparisons. (b) Heatmap showing expression profiles of DEGs from hierarchical clustering analysis. (c) Hallmark gene set annotations and (d) MBM metaprograms for the DEGs, with average expression values plotted for genes contributing to these gene sets.

To further explore inter individual tumor heterogeneity, we extended our analysis to the transcriptome, performing DE analysis to compare mRNA expression patterns across patient samples. Untreated tumor samples exhibited significant differences when compared to therapy-exposed tumors. DE analyses identified 2037 genes with significant differences (FDR>0.001, log2FC > ±2) between treated and untreated samples. Among these, 651 genes were also detected in the proteome, with 178 showing significant mRNA-protein correlations (Fig. 8a). Similar to the proteome-level findings, hierarchical clustering of DEGs revealed distinct mRNA expression profiles for each tumor, with P2 and P4 clustering together **—** mirroring results from proteomics analysis (Fig. 8b).

To further dissect transcriptional heterogeneity, we analyzed the average mRNA expression profile of DEGs annotated by hallmark gene sets, grouped into eight key biological processes: development, DNA damage, immune response, cellular component organization, metabolism, stress response, proliferation, and signaling (Fig. 8c). Treated tumors displayed notable up- or down-regulation of gene sets associated with development, immune responses, and signaling pathways. Additionally, we evaluated DEGs based on MBM metaprograms to identify coherent mechanisms driving tumor heterogeneity (Fig. 8d). This analysis aligned with prior findings from ST and bulk proteome data, revealing distinct distributions of cell types **—** such as myeloid cells, CAFs, immune cells, and malignant cells **—** within each tumor. Furthermore, it highlighted the enrichment of key tumor-regulating pathways, including IFN-γ, PI3K-Akt-mTOR, EMT, inflammation, and innate immune responses.

## Discussion

Understanding the spatial and functional heterogeneity within the TME in MBM is critical for predicting treatment responses and overcoming resistance in ICIs. This study provides an in-depth exploration of individualized proteogenomic profiles using a comprehensive multiomics approach, contributing to a more nuanced view of patient-specific biology in MBM. We have integrated ST, multi-region bulk exome sequencing, proteomics, and transcriptomics to simultaneously capture both intertumor heterogeneity and ITH. The number of DEPs and DEGs varied both across patient tumors and within individual tumors, underscoring the complex and dynamic nature of MBM, shaped by both treatment exposure and intrinsic tumor characteristics. Our findings revealed that MBM tumors exhibit significant ITH at both the molecular and cellular levels. For example, in patient P1, ST identified localized immune cell infiltration and EMT markers, while bulk proteomics highlighted distinct protein expression patterns across different tumor regions. Similarly, mutational profiles varied within individual tumors, indicating complex ITH. On the intertumor level, immunotherapy-treated patients (P2 and P4) showed enrichment in immune pathways like JAK-STAT, IFN-α/γ, and TNF-α signaling, while other tumors displayed elevated MAPK and EGFR signaling. These results demonstrate that even within a small cohort, there is substantial variability in MBM biology across patients, particularly in response to different treatments.

Bulk proteome and transcriptome profiling identified 7732 proteins and 18,784 protein-coding genes, with PCA demonstrating patient-specific clustering. We observed moderate mRNA-protein correlation, particularly in key MBM markers, with enrichment in pathways such as proliferation, IFN-γ, EMT, and immune responses, reflected in spatial ST analysis. Immunotherapy-treated patients showed distinct enrichment in EMT, oxidative phosphorylation, IFN-α/γ, T-cell, and DNA damage pathways. Both DEPs and DEGs aligned with immune cell types and melanoma-associated markers, emphasizing the consistency between transcriptomic and proteomic data in terms of central cellular characteristics.

Spatial-resolved transcriptomics data provided a detailed overview of cellular composition, with immune cell infiltration being significantly higher in treated patients. Cellular deconvolution confirmed the presence of tumor cells, CAFs, T cells, myeloid cells, and CNS components, consistent with ST data. CAFs and EMT signaling were prominent in patients P1 and P4, which is an interesting find taken their role in promoting brain metastasis and resistance to therapy [[36], [37], [38], [39]]. CAFs are increasingly recognized as critical cellular players in the cancer microenvironment, including brain metastasis and metastatic melanoma [26], promoting tumor progression and therapy resistance [28], through mechanisms such as epithelial-mesenchymal transition (EMT) regulation, extracellular matrix (ECM) remodeling, and immune evasion [26,34]. In this study, CAFs were identified among prominent cell types in the MBM microenvironment, especially in patients P1 and P4, where their spatial distribution correlated with elevated EMT signaling. Emerging studies also suggest that targeting CAFs or their secreted factors may improve the efficacy of ICIs and other cancer therapies [26]. Approaches that target CAF signaling pathways, such as TGF-β, FGF, or CXCL12, or disrupt CAF-tumor interactions, hold promise for enhancing the effectiveness of current treatments. Similarly, EMT represents a critical pathway associated with metastasis and treatment resistance, particularly in patients with elevated EMT signaling like P1 and P4. EMT blockers, such as those targeting E-cadherin, Snail, or ZEB1, could potentially reverse EMT, and sensitize tumors to immunotherapies and other treatments in MBM. Overall, this study supports the potential of targeting CAFs and EMT as strategies to overcome resistance and improve therapeutic outcomes in MBM patients.

In addition to immune and signaling pathway variability, we also examined melanocytic lineage programs and their potential association with immunotherapy response. Our multiomics data revealed variable activation of melanocytic programs across MBM tumors. MITF, a central regulator of melanocyte identity, showed high activity in patients P3 and P4 but was absent in P1, consistent with a dedifferentiated phenotype. Proteomic analyses confirmed the presence of melanogenesis-related proteins (e.g., TYRP1, MLANA) in certain tumors, although classical melanin biosynthesis genes (TYR, DCT) were not strongly expressed. These trends are visualized in the patient-level melanogenesis gene expression profiles shown in Figure S14. Despite previous reports suggesting that active melanogenesis may contribute to immune evasion and therapy resistance through immunosuppressive microenvironments [[40], [41], [42]], our data does not show a consistent association between melanogenesis activation and clinical outcomes. For example, patient P4 exhibited higher melanogenesis activity and longer survival than P2, despite both receiving immunotherapy; however, this may also be influenced by BRAF V600E mutation status. Additionally, immune-related markers such as HLA-A, VEGFA, TGFB1, and HIF1A showed no consistent trend relative to melanogenesis status. Interestingly, studies [43] have reported higher melanogenesis gene expression in immunotherapy responders. Taken together, while our samples span the spectrum from melanotic to amelanotic tumors, we find no conclusive evidence linking melanogenesis activation to immune response or survival in MBM.

Given the complexity of tumor-host interactions and the possibility of extratumoral regulatory influences, we next explored whether MBM tumors might engage broader systemic mechanisms, including neuroendocrine signaling pathways. Recent study [44] have suggested that malignant tumors, including melanomas, may disrupt systemic homeostasis by producing neuroendocrine mediators such as corticotropin-releasing hormone (CRH), proopiomelanocortin (POMC)-derived peptides (e.g., ACTH), and melatonin, thereby hijacking the host’s neuroendocrine network to favor tumor progression. To examine this hypothesis, we compared stromal regions (<25 % malignant cell content) with tumor-dominant areas (>75 % malignant content) across our spatial transcriptomic data. However, we did not detect expression of several key neuroendocrine genes implicated in this model, including CRH, GHRH, TSHB, TH, DBH, TPH2, CALCA, TAC1, and CYP11A1 (see Figure S15). While some signaling molecules such as IL-6 were detectable, their expression did not correlate with clinical outcomes, and they were more prominent in non-tumor cell compartments. Moreover, due to the lack of matched healthy brain tissue and extracranial controls, we cannot infer systemic neuroendocrine impact. Therefore, while the concept of tumor-induced neuroendocrine modulation is compelling, our data does not support its presence in MBM and such mechanisms remain beyond the scope of this study.

Overall, while our study provides valuable insights into the molecular and cellular landscape of MBM, few limitations must be acknowledged. First, the small sample size of four patients restricts the generalizability of the findings to the broader melanoma population, limiting the ability to draw definitive conclusions. The regional selection of tumor samples may also introduce sampling bias, as they were derived from specific areas of the metastases. Additionally, the use of fresh frozen samples for ST analysis poses a challenge, as such samples are not routinely collected in clinical settings, limiting the method's broader applicability. Moreover, the lack of validation in an independent cohort or through additional experimental approaches restricts the ability to confirm the robustness of the identified molecular signatures. Future studies should aim to validate these findings in larger, independent cohorts to assess the clinical relevance of the identified markers and pathways. Finally, while the study identifies potential therapeutic targets, these require validation in diverse cohorts and clinical trials to establish their utility in treatment strategies for MBM.

In conclusion, our study demonstrates the utility of multiomics analysis in uncovering patient-specific molecular alterations and deepening our understanding of tumor heterogeneity, cellular composition, and key signaling pathways in melanoma brain metastases (MBM). By integrating spatial transcriptomics with bulk genomic and proteomic profiling, we provide a comprehensive spatial, transcriptomic, and proteogenomic view of the MBM tumor landscape. This approach revealed pronounced inter- and intra-tumoral heterogeneity, with distinct molecular and cellular features shaped by prior treatment exposures. Immunotherapy-treated tumors (P2, P4) showed enriched immune and inflammatory signaling, while untreated or radiotherapy-exposed tumors (P1, P3) exhibited prominent stromal and EMT-associated programs. Importantly, proteomic and transcriptomic data were mutually consistent for key melanoma markers, and spatial analyses confirmed the architecture of immune-rich versus CAF-rich regions. Although melanocytic lineage activity varied across tumors, it did not consistently correlate with clinical outcomes, underscoring the complexity of immune resistance in MBM. Overall, these findings offer insights that may guide future efforts to identify novel therapeutic targets—particularly those involving the tumor stroma and EMT pathways—and improve treatment strategies for this challenging condition.

## Data availability statement

All data generated or analyzed during the study, are either included in the article or uploaded as supplementary data. Bulk proteomics data have been uploaded to the PRIDE repository via ProteomeXchange with accession numbers PXD055089, PXD055170, and PXD055172. Bulk mRNA data was uploaded on GEO portal with accession number GSE275731. DNA data is provided as part of supplementary information. The ST data is uploaded at https://doi.org/10.5281/zenodo.13941926. The pipeline developed for ST analysis is available at https://github.com/jana-rajova/Mmint.

## CRediT authorship contribution statement

**Nidhi Sharma:** Writing – review & editing, Writing – original draft, Visualization, Validation, Resources, Methodology, Investigation, Formal analysis, Data curation. **Jana Rájová:** Writing – review & editing, Visualization, Validation, Formal analysis. **Georgios Mermelekas:** Methodology, Data curation. **Kim Thrane:** Methodology, Data curation. **Joakim Lundeberg:** Validation, Resources. **Alia Shamikh:** Validation, Methodology. **Sofi Vikström:** Resources, Validation. **Haris Babačić:** Validation, Methodology. **Margret Jensdottir:** Validation, Resources. **Janne Lehtiö:** Validation, Resources. **Maria Pernemalm:** Writing – review & editing, Supervision, Project administration, Funding acquisition. **Hanna Eriksson:** Writing – review & editing, Supervision, Project administration, Funding acquisition, Conceptualization.

## Declaration of competing interest

The authors declare that they have no known competing financial interests or personal relationships that could have appeared to influence the work reported in this paper.

## Appendix Supplementary materials

Image, application 1
