# Supplementary material for: In-depth patient-specific analysis of tumor heterogeneity in melanoma brain metastasis: Insights from spatial transcriptomics and multi-region bulk sequencing
Source: Transl Oncol. 2025 Jul 15;59:102468. doi: 10.1016/j.tranon.2025.102468 (PMC12284558; doi:10.1016/j.tranon.2025.102468)
Supplement: Supplementary file 1 [file mmc1.pdf]

Supplementary information for "In-Depth Patient-specific Analysis of Tumor Heterogeneity in Melanoma Brain Metastasis: Insights from Spatial Transcriptomics and Multi-Region Bulk Sequencing"

Nidhi Sharma[1,4,*], Jana Rájová[2], Georgios Mermelekas[1,4], Kim Thrane[3,4], Joakim Lundeberg[3,4], Alia Shamikh[1,5], Sofi Vikström[1], Haris Babačić[1,4], Margret Jensdottir[7,8], Janne Lehtiö[1,4], Maria Pernemalm[1,4,#,*] and Hanna Eriksson[1,6,#,*]

[#]Shared last authors, *Corresponding authors

[1]Department of Oncology-Pathology, Karolinska Institute, 171 77 Stockholm, Sweden.

[2]Molecular Neuromodulation, Department of Experimental Medical Science, Lund University, 221 00 Lund, Sweden.

[3]Department of Gene Technology, KTH Royal Institute of Technology, 114 28 Stockholm, Sweden.

[4]Science for Life Laboratory, Tomtebodavägen 23, 171 65 Solna, Sweden.

[5]Clinical pathology and Cancer diagnostic Center, 171 76 Solna, Sweden.

[6]Theme Cancer, Skin Cancer Center, Karolinska University Hospital,171 77 Solna, Sweden.

[7]Department of Clinical Neuroscience, Karolinska Institutet, 171 77 Stockholm, Sweden.

[8]Department of Neurosurgery, Karolinska University Hospital, 171 76 Solna, Sweden.

Table S1. Synoptic pathology features of primary melanomas (P1-P4).

Patient (P)	Year of primary melanoma diagnosis	Histopathological features of the primary melanoma
P1	2007	*
P2	2015	Breslow thickness of 3.3 mm, Clark level: IV, ulceration present, mitotic rate 3-4 mitotic figures/mm^2, regression absent. Histological subtype: could not be determined. Radically excised with 4 mm margin.
P3	2018	**
P4	2004	Breslow thickness of 0.5 mm, Clark level: II, ulceration absent, lymphovascular invasion absent. Radically excised with 1.5 mm margin

* The patient underwent surgical treatment in a different region of Sweden, and histopathological data regarding the primary tumor are unavailable

** The patient was diagnosed with central nervous system metastases at initial presentation. As the primary tumor was not surgically resected, histopathological classification could not be performed.

Table S2: Overall Survival (OS) and Progression-Free Survival (PFS) for Each Patient. OS is the time from the operation for melanoma brain metastases (MBM) until death from any cause. PFS is defined as the time from the operation until disease progression or death from any cause.

Patient	OS (days)	OS (years)	PFS (days)	PFS (years)
P1	13	0.036	13	0.036
P2	110	0.30	84	0.23
P3	63	0.17	63	0.17
P4	222	0.61	111	0.30

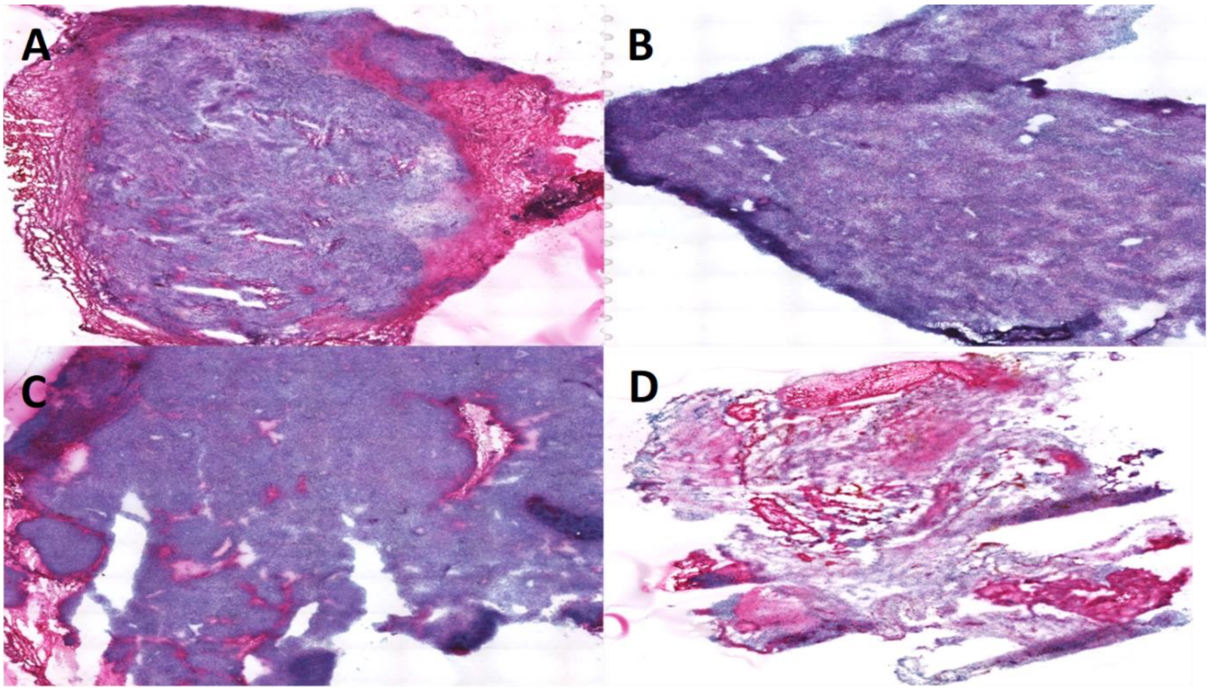

Figure S1 Hematoxylin and eosin (H&E) staining of frozen sections from melanoma brain metastases (MBM). (a) Sample from patient 1, showing predominantly tumor cells with surrounding glial tissue and peripheral red blood cells. (b) Sample from patient 2, consisting mainly of tumor cells with sparse intervening stroma. (c) Sample from patient 3, displaying primarily tumor cells with limited stroma and red blood cells in the periphery. (d) Sample from patient 4, showing tumor cells with necrosis, hemorrhage and sparse glial tisuue.

Histological description of the tumor

P1: Microscopically atypical tumor cells with pleomorphism and prominent nucleoli can be seen growing in a solid pattern with mitotic figure. Tumor necrosis is present. The tumor is surrounded by glial tissue with bleeding, and fibrosis.

P2: Microscopically atypical tumor cells with pleomorphic cells and prominent nucleoli growing in a solid pattern with mitotic figures and focal pigment within the tumor. The tumor is surrounded by glial tissue.

P3: Microscopically atypical tumor cells with pleomorphism and prominent nucleoli with mitotic figures and focal pigment within the tumor. Lymphovascular invasion can be seen and focal bleeding.

P4: Microscopically atypical tumor cells with pleomorphism and prominent nucleoli can be seen growing in between focal myxoid areas. Extensive tumor necrosis is present. Focal glial tissue can be seen.

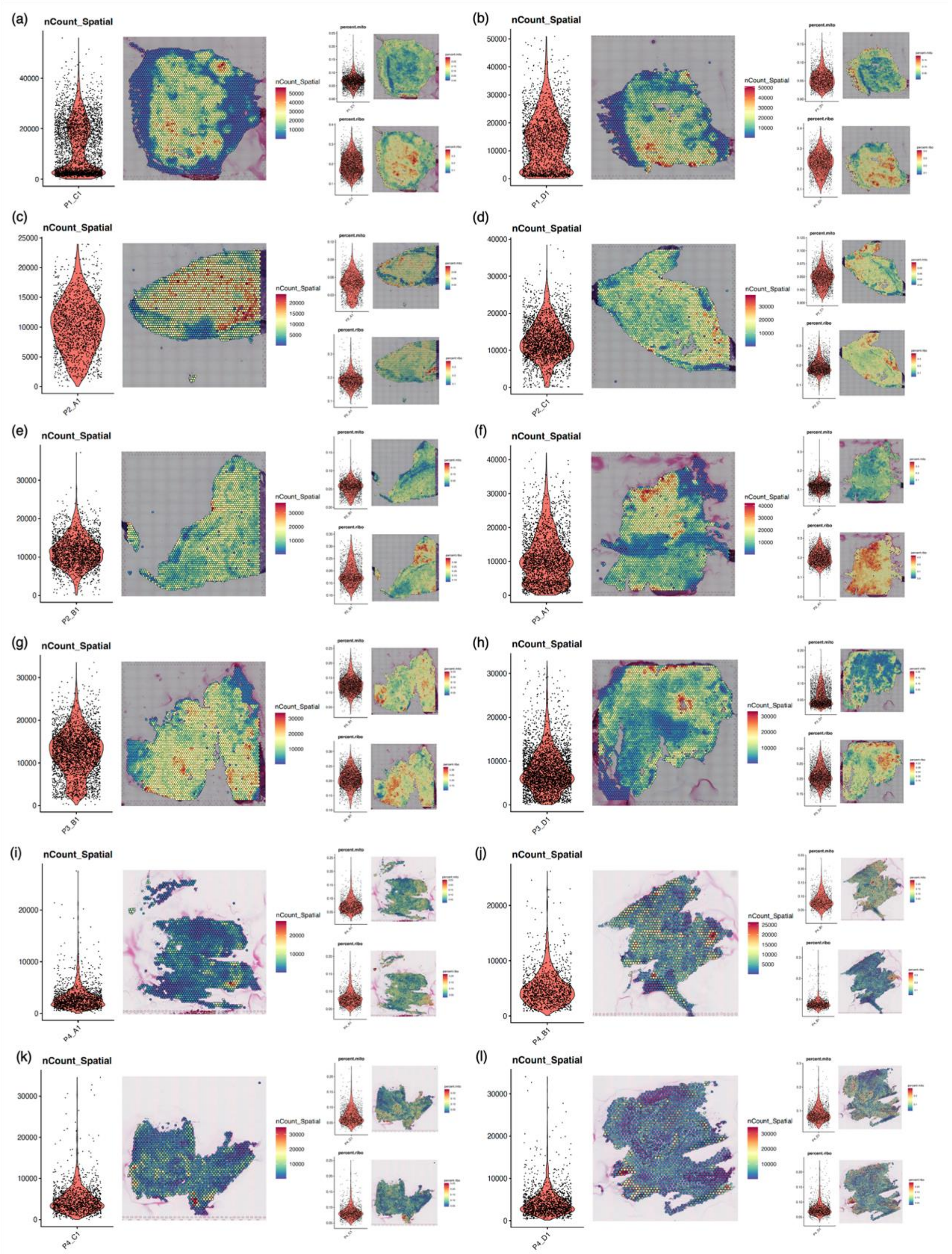

Figure S2 Quality control and summary of Visium ST data. (a-i) Violin plots and spatial visualization of distribution of UMI counts, and percentage of mitochondrial and ribosomal genes detected in each sample.

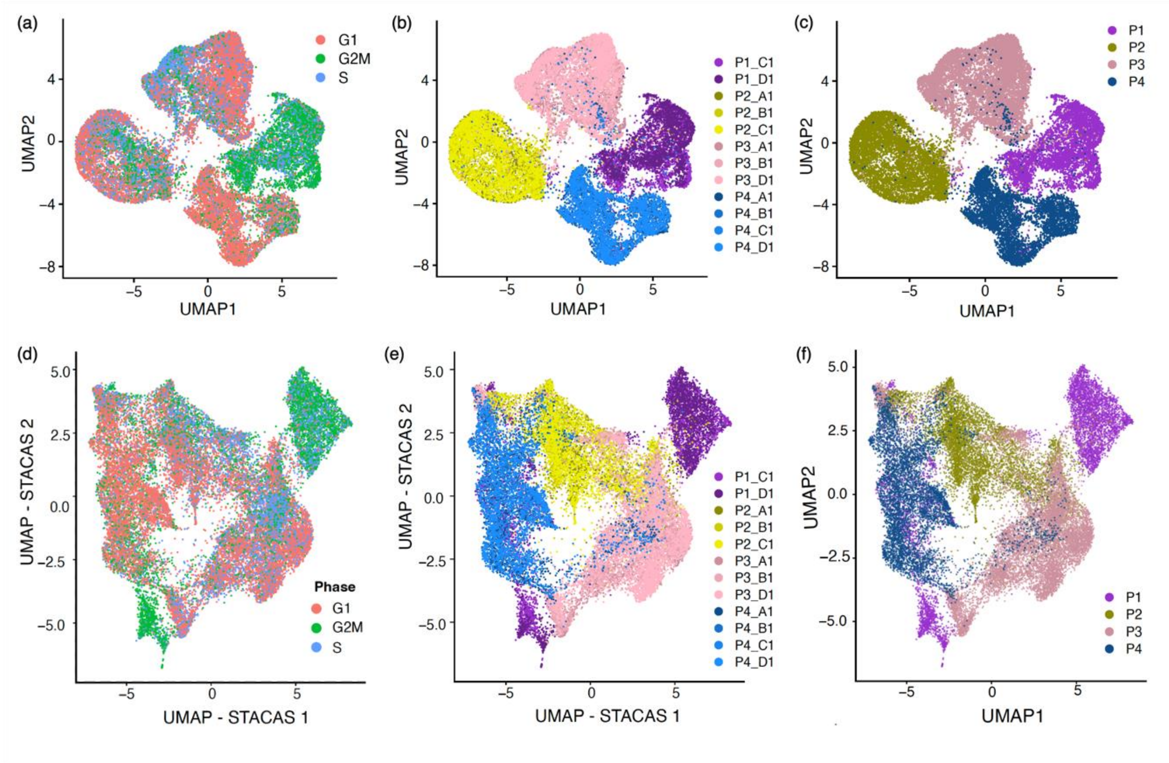

Figure S3 Comparison of the batch corrected and non-batch corrected Visium ST dataset. (a-c) UMAP plots of features from all samples colored based on cell cycle phase, patient and all individual sample replicates. (d-e) Comparison of distribution of features from patients before and after batch correction with STACAS.

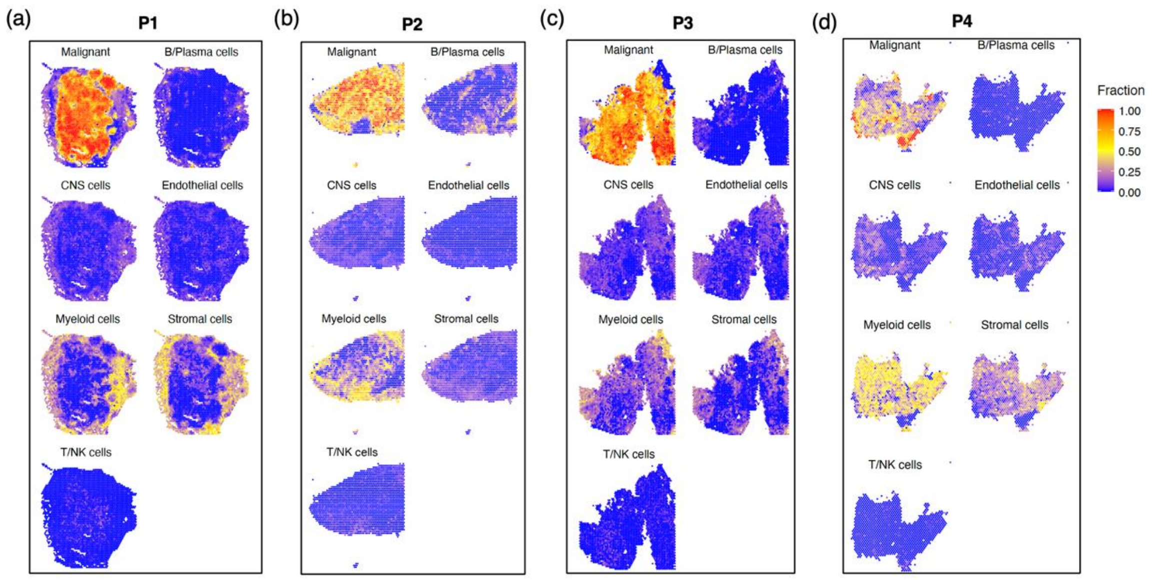

Figure S4 Cell type deconvolution of ST data. (a-d) Spatial plots showing the distribution of major cell type classes in each patient sample.

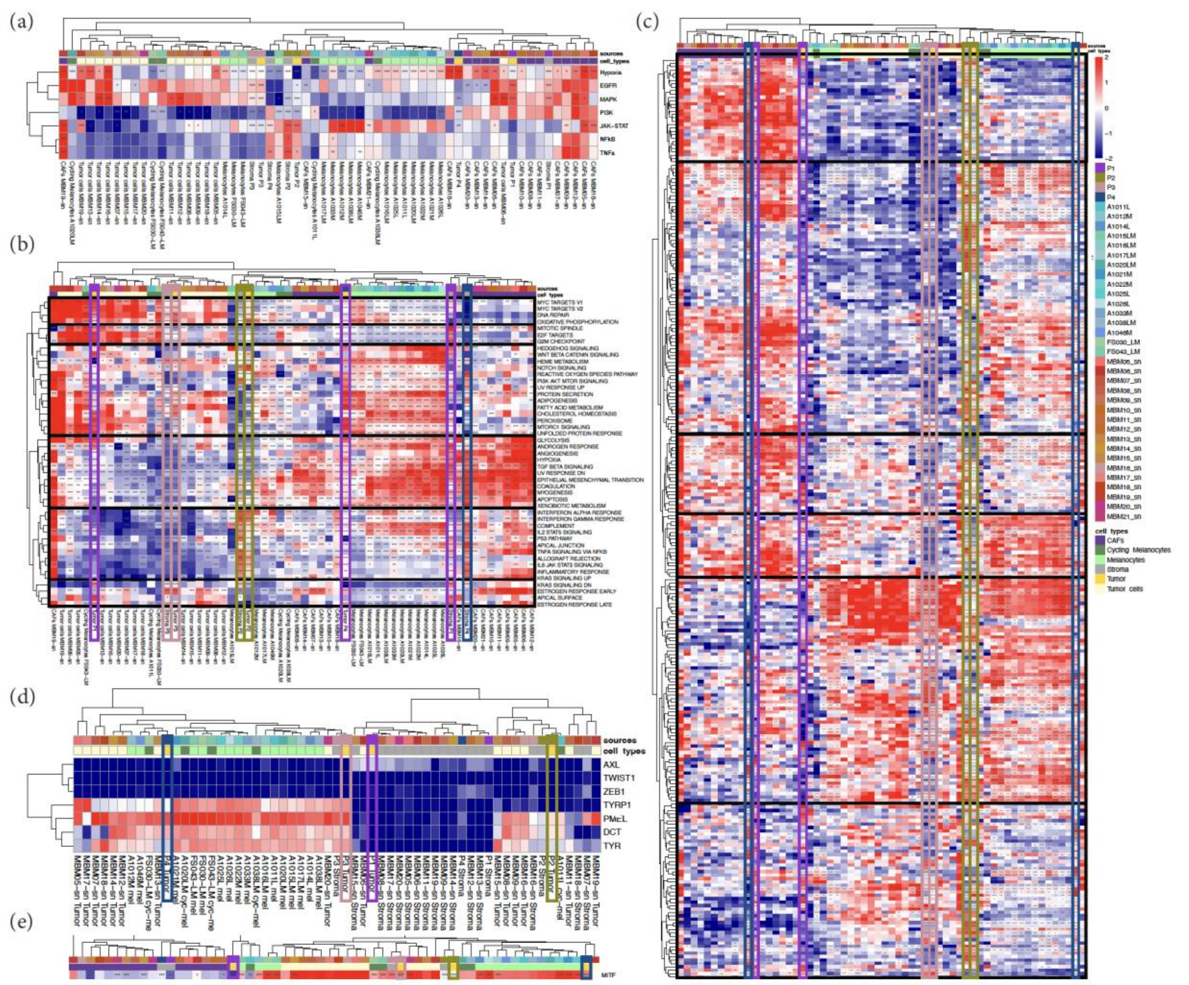

Figure S5 Validation of MBM transcriptomic signatures through comparison with normal melanocytes and naïve MBM profiles. (a) Scores for activation of PROGENy pathways, (b) scores for activation of HALLMARK pathways, (c) scores for activation of TFs according to the CollectTRI database, (d) expression values for melanocyte marker genes and invasion-associated genes and (e) detailed view of estimated MITF activation for normal melanocytes, naive melanoma brain metastases and MBM patient group (P1- P4) from our study.

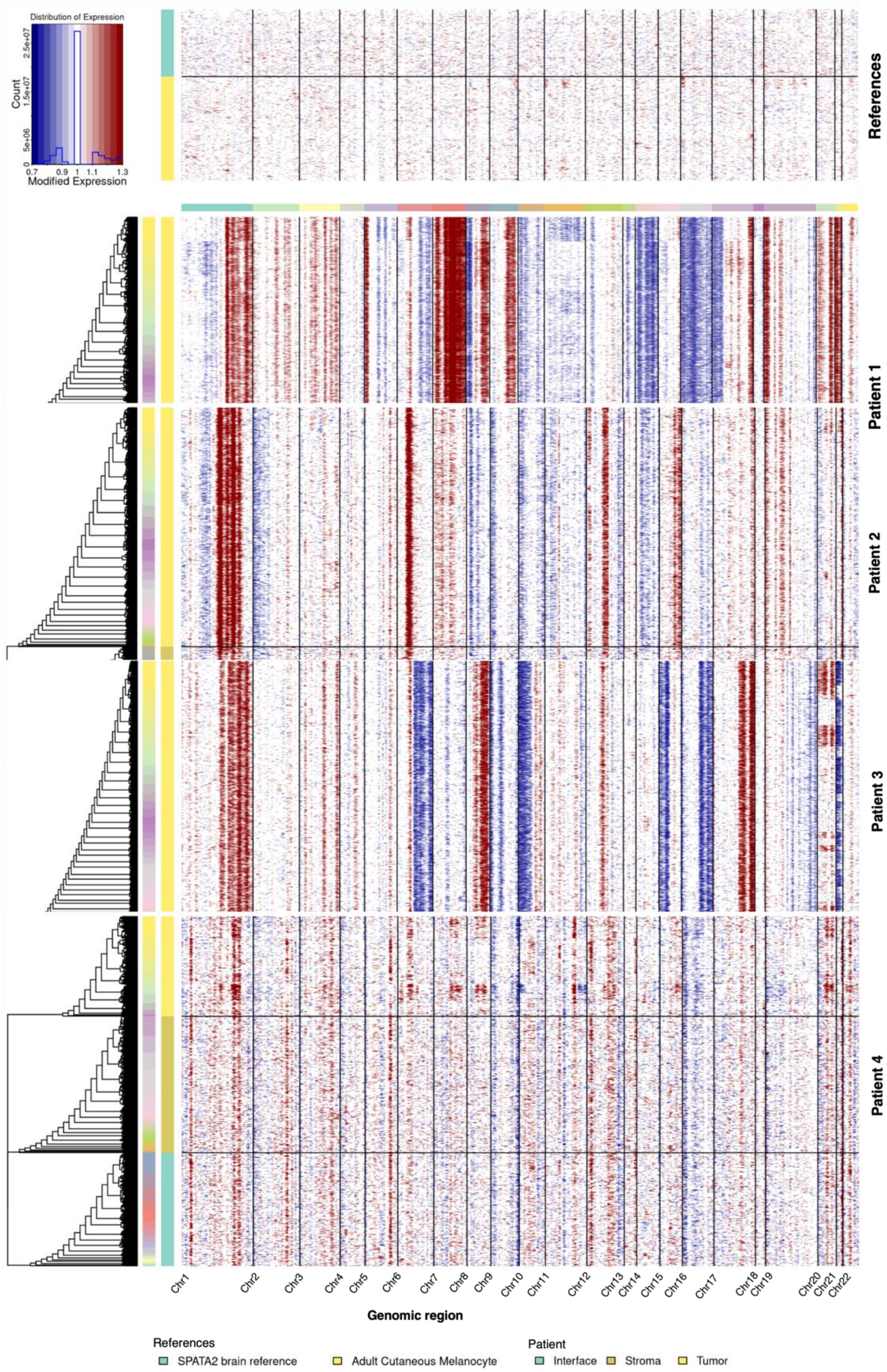

Figure S6 Estimation of copy number variations (CNV) using reference data from healthy melanocytes and brain cells with inferCNV illustrating the relative expression intensities across each chromosome. Chr., chromosome.

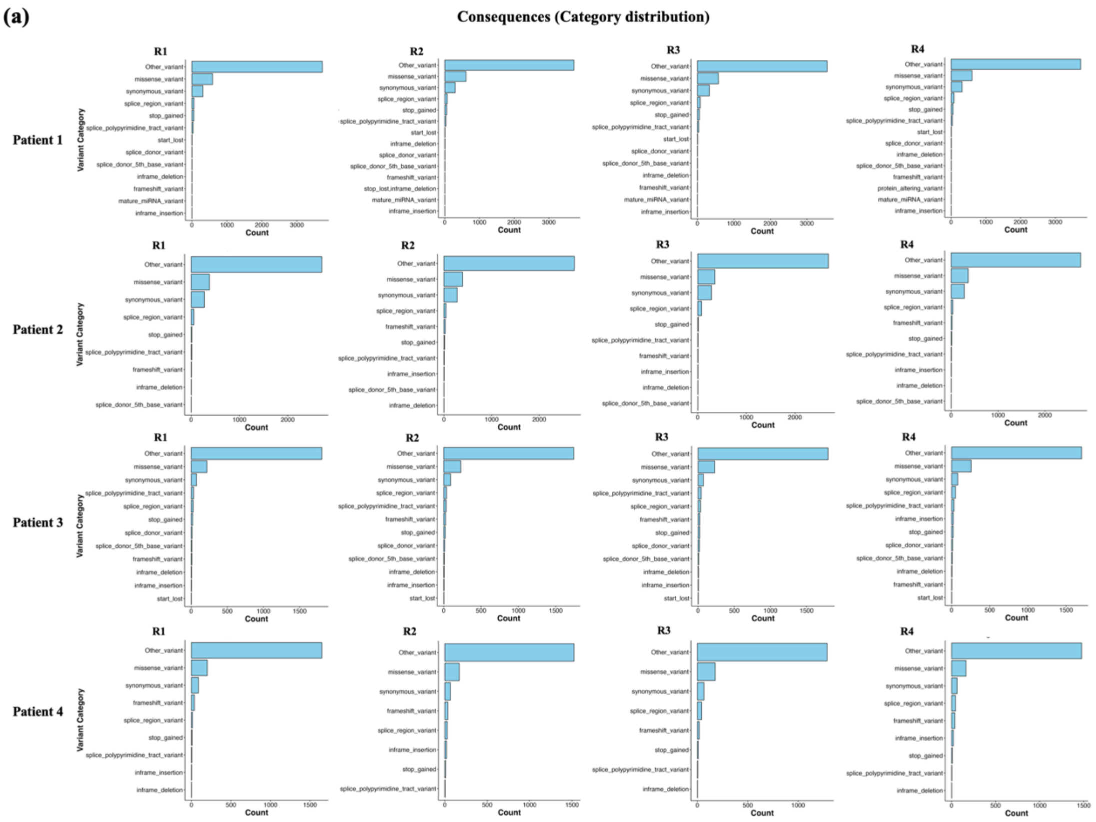

Figure S7 Identification of MBM tumor variations using bulk tumor exome sequencing. (a) The bar plots showing distribution of consequences including synonymous and different types of non-synonymous mutations detected in multiple tumor regions of each patient tumor tissue. Here, we used filtered variations data for increased clinical significance, by filtering out variants that are too common to be pathogenic using the low max_AF cutoffs of 0.0001 (0.01%).

(a)

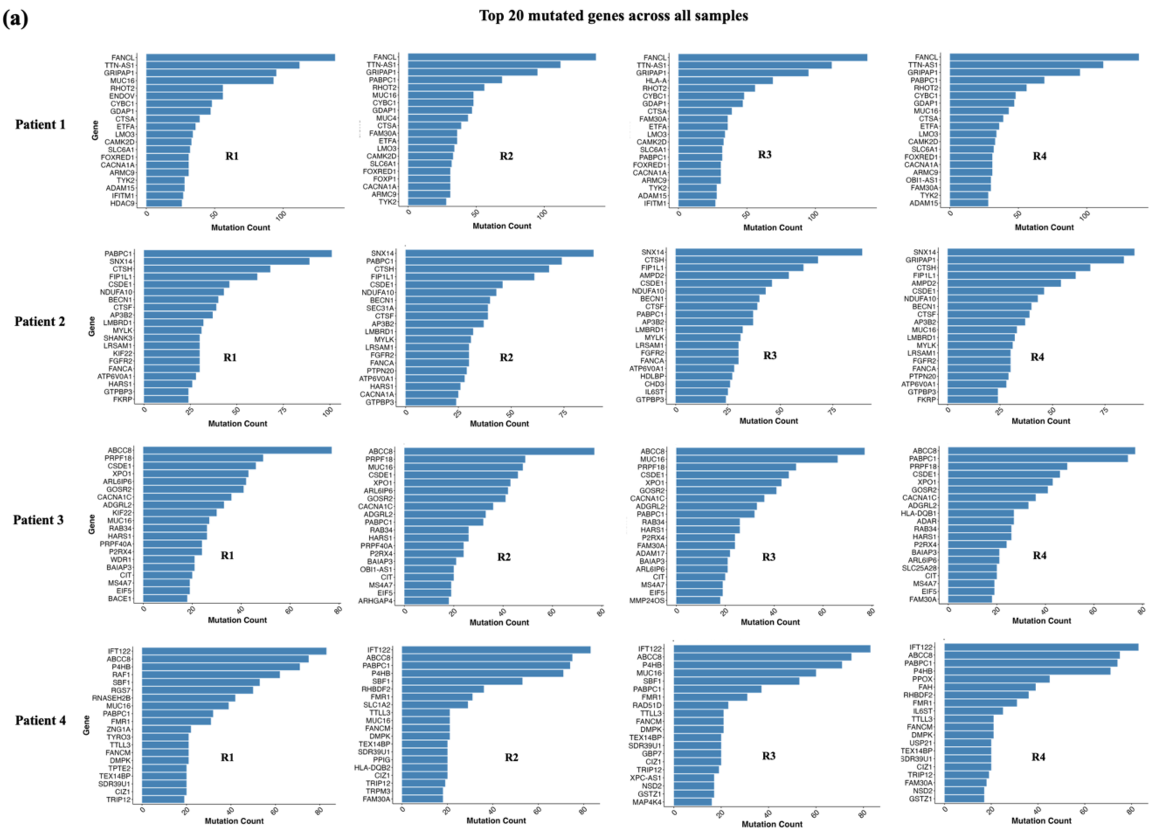

Figure S8 Mutation frequencies and significantly mutated genes identified in each tumor. (a) The bar plots showing the top 20 mutated genes observed in multiple tumor regions (R) of each patient (P) tumor tissue. The mutated genes are plotted based on the total number of variations detected per gene. Here, we used filtered variations data for increased clinical significance, by filtering out variants that are too common to be pathogenic using the low max_AF cutoffs of 0.0001 (0.01%).

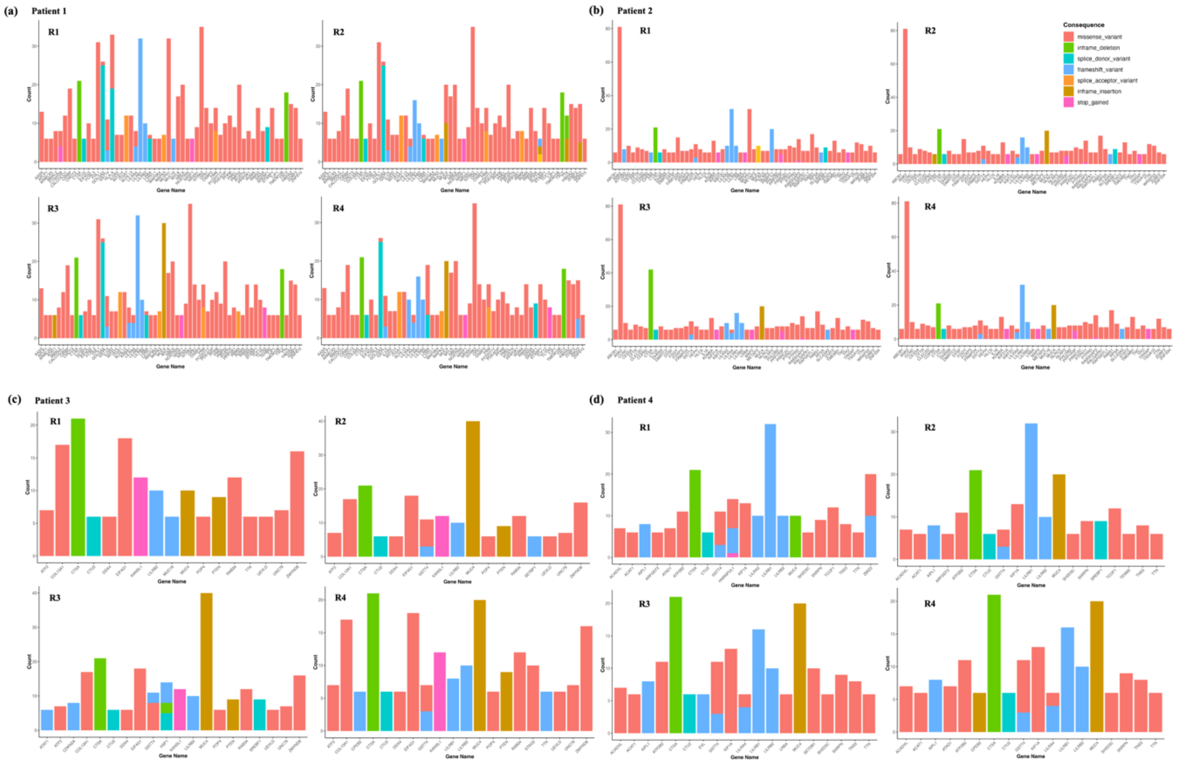

Figure S9 Potential novel gene variants identified in MBM. (a) The bar plots showing the number and types of potential novel gene variants found in multiple tumor regions (R) of each patient (P) tumor. The legend shows bar color for each consequence category. Out of the total novel variations identified, here, the plots are displaying novel variants with Impact status either "moderate" or "high", and variant frequency of > 5 were considered for potential novel variant analysis. Here, we used filtered variations data by excluding variants that are too common to be pathogenic using the low max_AF cutoffs of 0.0001 (0.01%).

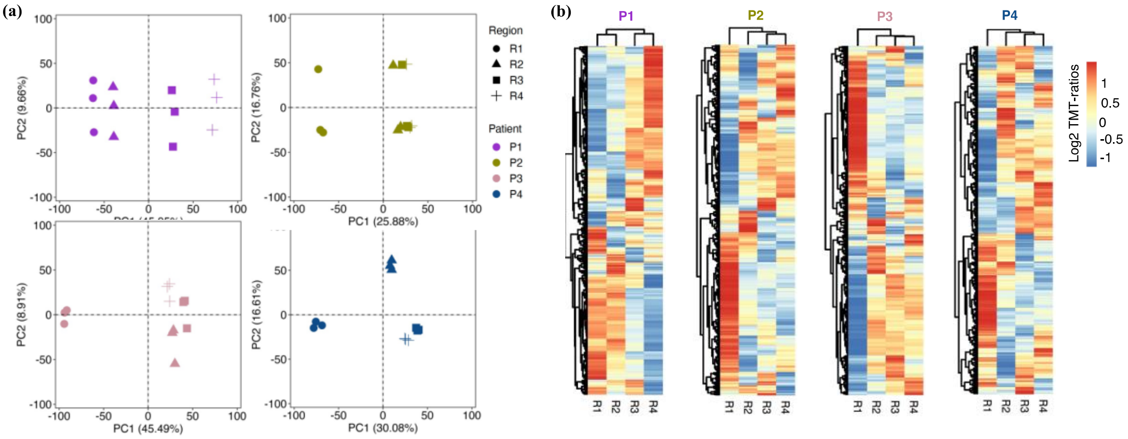

Figure S10 Multi-region bulk proteomics reveals distinct patterns of ITH in MBM. (a) PCA analysis shows distribution of variance observed between the multiple regions (R), and (b) heatmap representation shows expression patterns of total proteins identified in each patient tumor, using multi-region tissue proteome profiling.

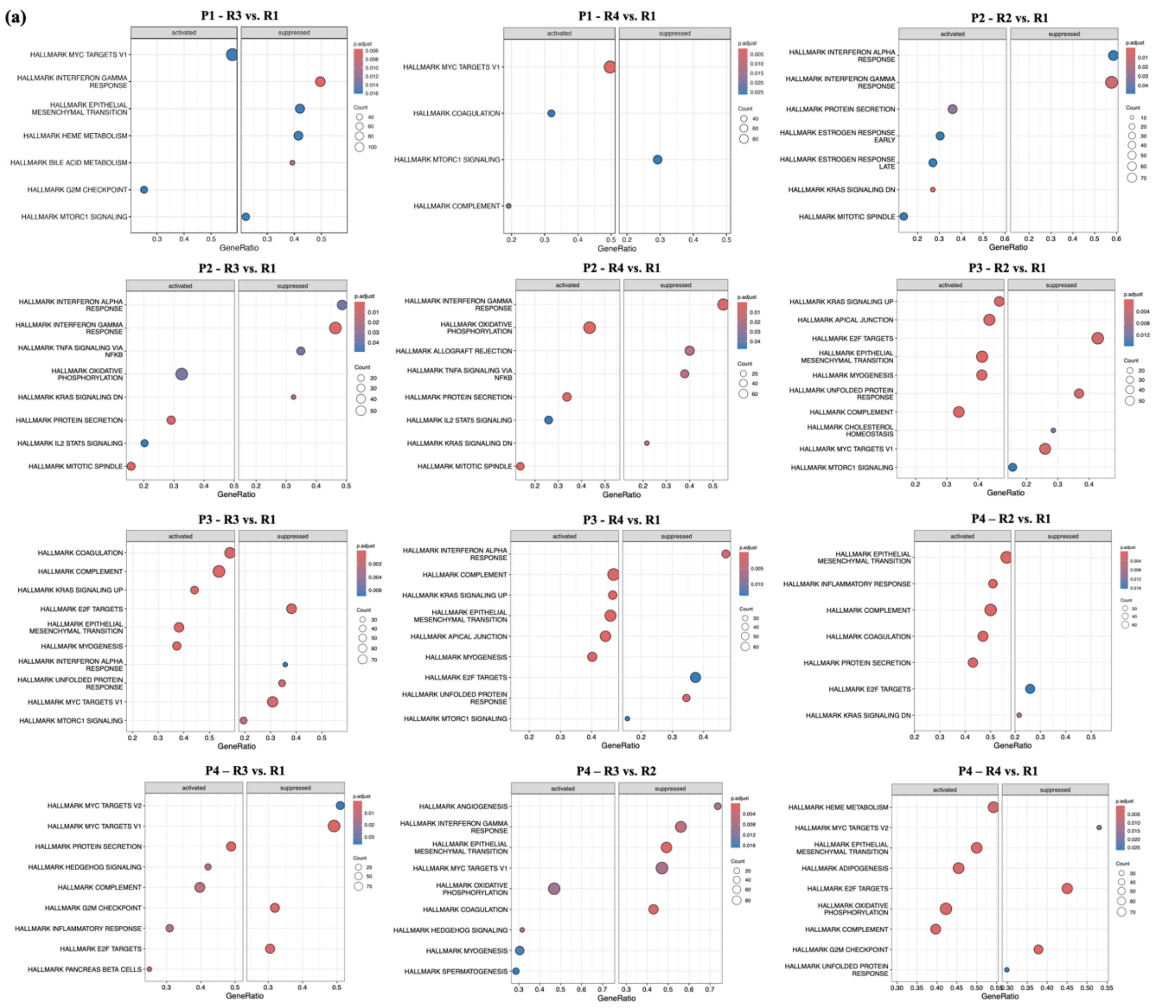

Figure S11 Intratumor heterogeneity observed within each patient tumor. Gene set enrichment analysis (GSEA) of DEPs identified for following tumor comparisons- (a) P1 vs. Others, (b) P2 vs. Others, (c) P3 vs. Others and (d) P4 vs. Others. Significantly enriched hallmark pathways based on GSEA. Here, the circle colour and size corresponds to adjusted p-values and gene count, respectively.

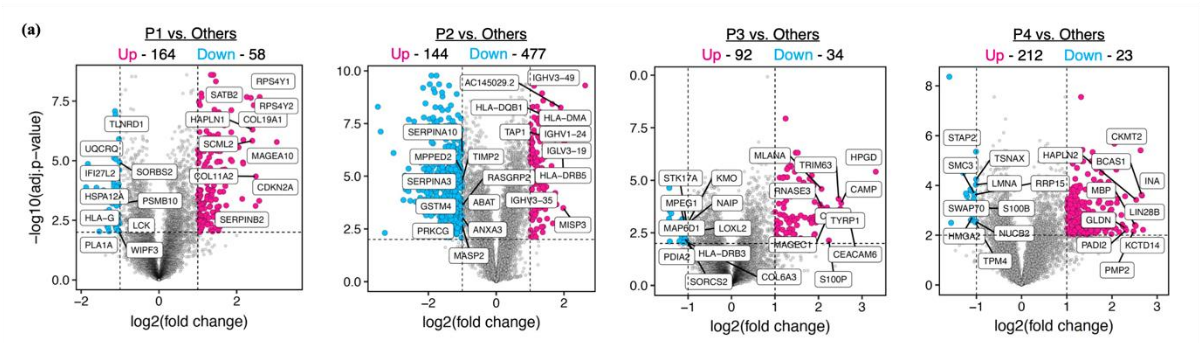

Figure S12 Inter-patient tumor heterogeneity in the MBM proteome. (a-d) Volcano plots depicting significantly altered protein abundances across individual patient tumors in comparison to others. Up-regulated DEPs are highlighted in pink, down-regulated DEPs in blue, and non-significant proteins in grey.

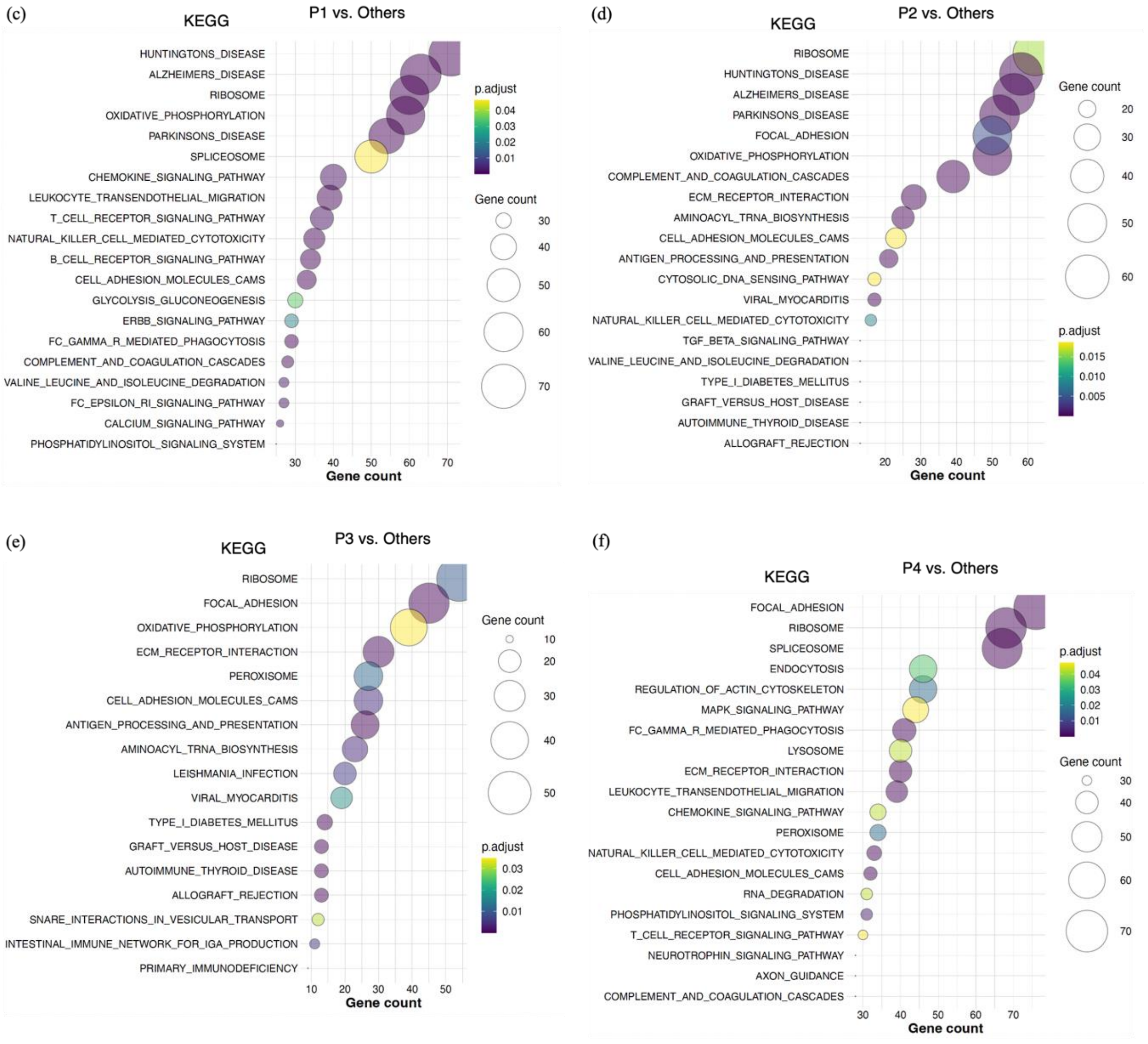

Figure S13 Gene set enrichment analysis (GSEA) for DEPs identified for following tumor comparisons- (a) P1 vs. Others, (b) P2 vs. Others, (c) P3 vs. Others and (d) P4 vs. Others. Significantly enriched KEGG pathways based on GSEA. For KEGG, the circle colour and size corresponds to adjusted p-values and gene count, respectively.

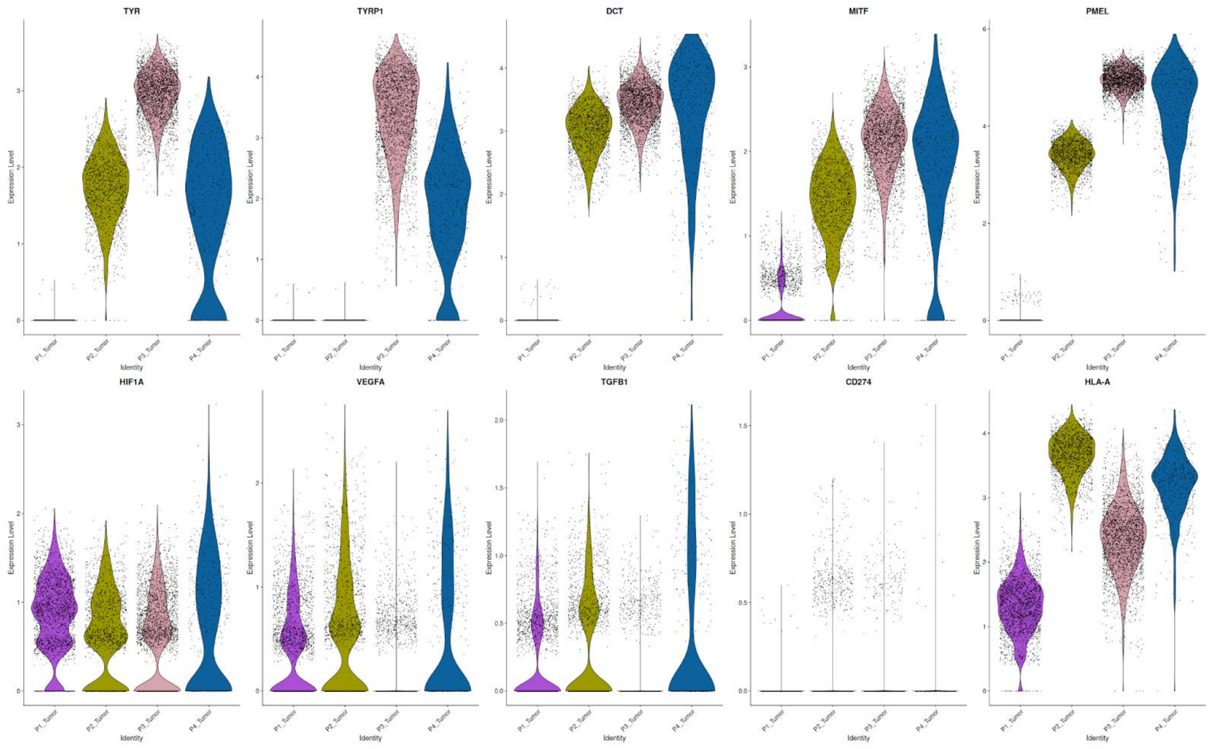

Figure S14 Expression of melanogenesis genes (TYR, TYRP1, DCT, MITF, PMEL) and genes involved in immunosuppressive micro-environment (HIF1A, VEGFA, TGFB1, CD274, HLA-A) in the tumor areas (>75% malignant cells in a feature).

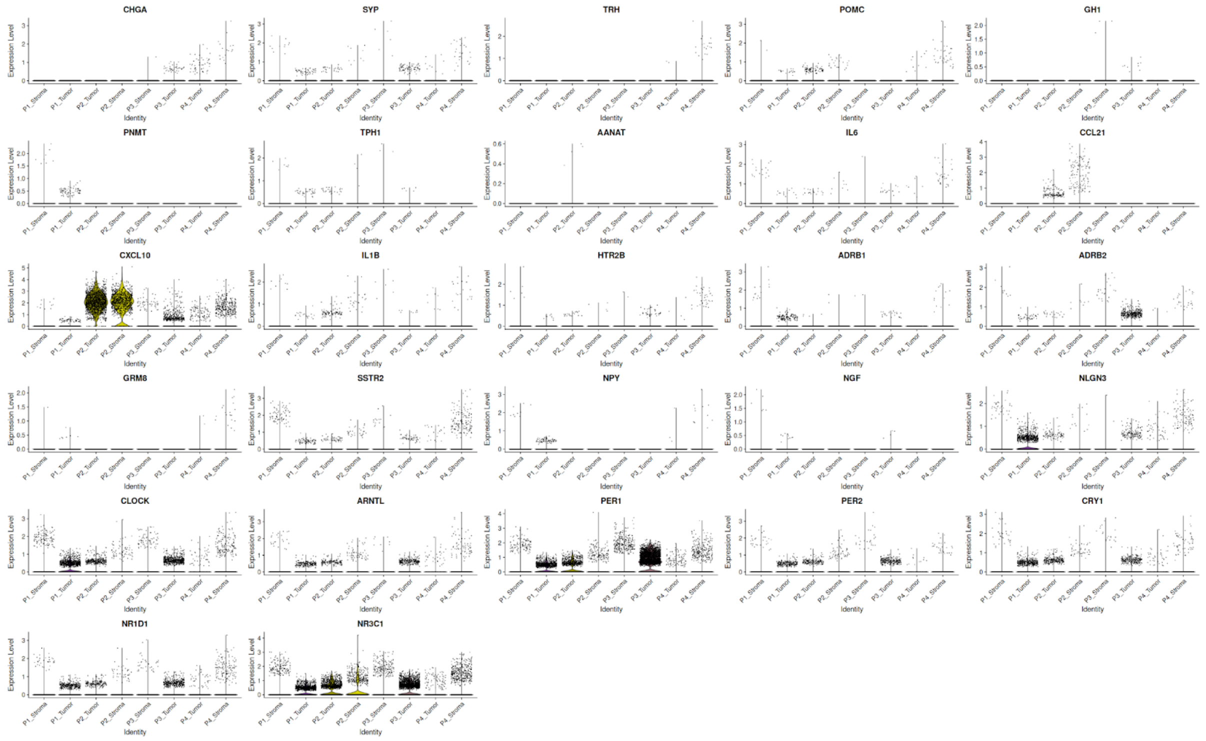

Figure S15 Expression of neurotransmitter genes in the tumor areas (>75% malignant cells in a feature) compared to the stromal areas (25%< malignant cell proportion).
